# Success of Montreal Protocol Demonstrated by Comparing High-Quality UV Measurements with “World Avoided” Calculations from Two Chemistry-Climate Models

**DOI:** 10.1038/s41598-019-48625-z

**Published:** 2019-09-03

**Authors:** Richard McKenzie, Germar Bernhard, Ben Liley, Patrick Disterhoft, Steve Rhodes, Alkiviadis Bais, Olaf Morgenstern, Paul Newman, Luke Oman, Colette Brogniez, Stana Simic

**Affiliations:** 1 National Institute of Water & Atmospheric Research (NIWA), Lauder, New Zealand; 2 Biospherical Instruments Inc., San Diego, CA USA; 3 CIRES-University of Colorado, Boulder, CO USA; 4 NOAA Global Monitoring Division – Radiation Group, Boulder, CO USA; 5 Bureau of Meteorology, Melbourne, Australia; 6 Aristotle University of Thessaloniki, Thessaloniki, Greece; 7 National Institute of Water & Atmospheric Research (NIWA), Wellington, New Zealand; 8 NASA Goddard Space Flight Center, Greenbelt, MD USA; 9 Univ. Lille, CNRS, UMR 8515 - Laboratoire d’Optique Atmosphérique, F-59000 Lille, France; 10 Universität für Bodenkultur, Vienna, Austria

**Keywords:** Risk factors, Atmospheric chemistry, Atmospheric chemistry

## Abstract

The Montreal Protocol on Substances that Deplete the Ozone Layer has been hailed as the most successful environmental treaty ever (https://www.unenvironment.org/news-and-stories/story/montreal-protocol-triumph-treaty). Yet, although our main concern about ozone depletion is the subsequent increase in harmful solar UV radiation at the Earth’s surface, no studies to date have demonstrated its effectiveness in that regard. Here we use long-term UV Index (UVI) data derived from high-quality UV spectroradiometer measurements to demonstrate its success in curbing increases in UV radiation. Without this landmark agreement, UVI values would have increased at mid-latitude locations by approximately 20% between the early 1990s and today and would approximately quadruple at mid-latitudes by 2100. In contrast, an analysis of UVI data from multiple clean-air sites shows that maximum daily UVI values have remained essentially constant over the last ~20 years in all seasons, and may even have decreased slightly in the southern hemisphere, especially in Antarctica, where effects of ozone depletion were larger. Reconstructions of the UVI from total ozone data show evidence of increasing UVI levels in the 1980s, but unfortunately, there are no high-quality UV measurements available prior to the early 1990s to confirm these increases with direct observations.

## Introduction

Concern about ozone depletion arose primarily because of its potential to increase UV-B radiation, and the consequent effects on health and the environment^1^. Observations of unexpected springtime decrease in stratospheric ozone over Antarctica^2^ — commonly referred to as the “ozone hole” — led to the rapid adoption of the Montreal Protocol on Substances that Deplete the Ozone Layer in 1987^3^. It has been shown that without this treaty and its subsequent Amendments and Adjustments, ozone holes would by now also be occurring over the North Pole, resulting in highly elevated UVI in the Arctic^4^. Even at mid-latitudes, the UVI would by now have increased markedly, and would more than double at some latitudes by the middle of the 21^st^ century^5–7^. However, due to the success of the Montreal Protocol, ozone decreases appear to have been brought under control^8^, though any effects on surface UV irradiances have not previously been confirmed by measurements.

We attempt to redress this issue by using an analysis of long-term UV measurements from instruments that meet the stringent requirements of the Network for the Detection of Atmospheric Composition Change (NDACC, www.ndacc.org).

## NDACC Measurements

The network was established in 1991 to ensure the highest quality monitoring of stratospheric properties that may be influenced by mankind^9^. Initially it was called the Network for the Detection of Stratospheric Change (NDSC) because the focus at that time was on ozone depletion. Its scope was broadened in 2005 to become NDACC in recognition of the increasing threats from climate change and an equal focus of the network on tropospheric composition. Spectral UV measurements had been part of the network since the mid-1990s when rigorous measurement criteria were established to ensure that changes in UV due to future ozone depletion would be detectable for ozone changes as small as 1%^10^. However, this detection target is extremely challenging and can be met only at wavelengths of less than 300 nm^11^. Here we focus on the UVI, which is a more relevant quantity from a health perspective. The UVI is a scaled version of the spectral irradiance weighted by the action spectrum for erythema (reddening from sunburn) in human skin^12^ and is widely used to disseminate UV information to the public^13^. In sunlight, the UVI is dominated by the contribution from wavelengths between 300 and 315 nm. NDACC protocols (https://www.ndsc.ncep.noaa.gov/organize/protocols/appendix6/) require a measurement uncertainty of less than 5% in this wavelength range. Given this uncertainty, changes in the UVI that are caused by a 4% change in total ozone become detectable^14^.

At the time the NDACC was established, the research community was mainly concerned about detecting increases in UV radiation due to the anticipated continuation of ozone depletion. However, because of the unprecedented success of the Montreal Protocol and its subsequent Adjustments and Amendments, the peak tropospheric chlorine loadings occurred before the turn of the century. Consequently, further ozone depletion and further UVI increases were not expected after that time. And over the longer term, a reduction in UVI is now projected due to ozone recovery^1^. But to date, there has been no clear demonstration of the effectiveness of the Montreal Protocol in curbing surface UV increases. This is partly due to a lack of high quality measurements prior to the early 1990s, but is also because of the compounding effects of other factors affecting UV radiation, including the effects of clouds and aerosols.

## Model Projections

We complement these measurements with projections from chemistry-climate models (CCMs), representing either the “World Avoided” or “World Expected” scenario. In the World Avoided scenario, the concentration of ozone-depleting substances continues unabated without being controlled by the Montreal Protocol. In contrast, the World Expected scenario simulates changes in ozone resulting from curbing ozone-depleting substances in full compliance with the Montreal Protocol^15^.

All CCM model projections must assume a Representative Concentration Pathway (RCP). The Intergovernmental Panel on Climate Change (IPCC) usually consider four pathways called RCP 2.6, RCP 4.5, RCP 6.0, and RCP 8.5, which are labelled according to the projected radiative forcing values in the year 2100 relative to pre-industrial values (+2.6, +4.5, +6.0, and +8.5 W/m^2^, respectively). Here we assume RCP 6.0 forcings^16^ for constituents other than the halogenated ozone-depleting substances (ODSs). In the World Avoided simulations, chlorinated or brominated source gases are assumed to increase by 3% per year from 1974 onwards. This rate of increase is modest compared to their actual rate of increase of 3.7 to 4% per year during the 1970s and 1980s before controls came into place^17^. In the World Expected simulations, all halogenated (chlorinated or brominated) ODSs follow the WMO (World Meteorological Organisation) A1 scenario^15^, which assumes compliance with the Montreal Protocol. Although now nine years old, more recent ODS scenarios differ only in minor regards from this scenario^8^.

## Data Analysis Method

We first calculate seasonal mean UVI values from the ozone fields projected by these CCM models to assess their long-term projections, out to the year 2100. We then compare these projected UVI values (out to year 2020) with corresponding measurements by NDACC instruments, as well as clear-sky UVIs calculated from measured total ozone columns. We finally assess whether observed UVIs differ substantially from those inferred from CCMs that either broadly follow the observed slow decline of stratospheric halogen, or alternatively, a scenario of increasing halogen.

The sites considered for analysis cover a wide range of latitudes in both hemispheres, including the tropics, mid-latitudes, and polar regions (Table 1).

**Table 1:** Details of sites included in the study. Sites shown with bold text have at least 20 years of data between 1996 and 2018.

Site	°Lat	°Long	Alt (m)	Years	Agency
Summit, Greenland	73 N	38 W	3202	2004–2017	BSI/NSF
**Barrow, Alaska**	**71** **N**	**157** **W**	**8**	**1991–2016**	**BSI/NSF**
Villeneuve-d’Ascq, France	51 N	3 E	70	2000–2018	LOA/UDL
**Hoher Sonnblick, Austria**	**47** **N**	**13 E**	**3106**	**1997–2018**	**BOKU**
Haute-Provence Obs., France	44 N	6 E	688	2009–2018	LOA/UDL
**Thessaloniki, Greece**	**41** **N**	**23 E**	**60**	**1990–2018**	**AUTH**
**Boulder, Colorado**	**40** **N**	**105** **W**	**1650**	**1998–2018**	**NIWA/NOAA**
San Diego, California	33 N	117 W	22	1993–2008	BSI/NSF
**Mauna Loa Obs., Hawaii**	**20** **N**	**156** **W**	**3400**	**1995–2018**	**NIWA/NOAA**
Saint-Denis, Réunion Island	21 S	55 E	80	2009–2018	LOA/UDL
Alice Springs, Australia	24 S	134 E	547	2003–2018	NIWA/BoM
Melbourne, Australia	38 S	145 E	110	2001–2018*	NIWA/BoM
**Lauder, New Zealand**	**45** **S**	**170 E**	**370**	**1990–2018**	**NIWA**
Ushuaia, Argentina	55 S	68 W	30	1988–2008	BSI/NSF
**Palmer Station, Antarctica**	**65** **S**	**64** **W**	**21**	**1990–2018**	**BSI/NSF/NOAA**
**Arrival Heights, Antarctica**	**78** **S**	**167 E**	**190**	**1989–2018**	**BSI/NSF/NOAA**
**South Pole**	**90** **S**	—	**2835**	**1991–2018**	**BSI/NSF/NOAA**

Abbreviations:AUTH, Aristotle University of Thessaloniki; BOKU Universität für Bodenkultur, Vienna (Austria); BoM, Bureau of Meteorology (Australia); BSI, Biospherical Instruments Incorporated (USA); LOA, Laboratoire d’Optique Atmosphérique (France); NOAA, National Oceanic and Atmospheric Administration (USA); NSF, National Science Foundation (USA); UDL, Université de Lille, Faculté des Sciences et Technologies, Villeneuve d’Ascq (France). *There were no data at Melbourne from 2003 to 2008.

Note that the Brewer spectrophotometer at Thessaloniki is not part of the NDACC network and does not meet all NDACC specifications, mainly because of the limited wavelength range of the instrument (280–365 nm). In the early part of the record (prior to mid-1993), only a single monochromator version of the Brewer instrument (range 290–330 nm) was used, which also had a poorer stray light rejection at shorter wavelengths. But for the purposes of this paper, which focuses on UVI, these limitations are less important. For the calculation of UVI, a model-derived correction factor is used to account for the missing part of the spectrum. The instrument at San Diego is also not part of NDACC; however, it is identical to the instruments used in Antarctica (South Pole, Arrival Heights, and Palmer Station). Similarly, the instrument at Melbourne is not yet part of the NDACC, although it is identical to those at Lauder, Mauna Loa, and Alice Springs.

We also note that at most sites, the data quality and frequency have both increased over time. For example, prior to 1996, data from Lauder were available only during periods when rain was not imminent, and for the following two years scans were disabled during (rare) rainy conditions. These limitations impart a potential clear-weather bias to early data at that site, particularly prior to 1996. Before 1997, measurements at the NSF (National Science Foundation) sites were taken only once per hour while spectral scans have been performed every 15 minutes in later years. Further analysis of data from a subset of sites suggests that these differences in the sampling protocol have virtually no effect on the results discussed in this study. At the Haute-Provence Observatory global irradiance measurements were available every 30 min prior to September 2010, and every 15 min after that date. At Villeneuve d’Ascq, scans are performed every 30 min.

We considered whether to use peak daily values, peak values during a specified time window (e.g., ±1 hour of local noon), or mean values over the noon period (e.g., ±0.5 hour of local noon). Variations in seasonal mean UVIs calculated from the three quantities agreed to within ±3%, which is smaller than the measurement uncertainty of ±5%^10^. By using peak values instead of mean values, cloud effects on seasonal averages are reduced and time-series derived from measurements therefore agree slightly better with our modelling results, which do not take attenuation from clouds into account. The following analysis is therefore based on seasonal means of the daily maximum UVI observed within ±1 hour of local noon (12:00 UTC at Greenwich; 00:45 UTC at Lauder, etc). Data gaps were treated as described by Bernhard^18^. In brief, single missing days were “filled in” by calculating the average of the UVI of adjacent days. Data gaps lasting for more than one day were corrected by taking climatological variations in SZA and ozone into account. If more than 30 days were missing within a 90-day period, seasonal means were excluded from further analysis.

Seasonal changes in UVI are large at mid- to high-latitudes. For latitudes poleward of 45°, UVI values in winter are less than 10% of the summer peaks. And in polar regions, the UVI reduces to zero during the polar night. Additionally, there are large day-to-day differences in UVI due to changing cloud conditions and types. To minimize these effects, we consider seasonal means of approximately 90 days (December–February, March–May, June–August, and September–November), which are also more relevant from an environmental perspective.

Measurements of UVI from the sites in Table 1 are compared with values calculated for clear skies for four different ozone scenarios:

1. ozone values from the NIWA/BS global total-column ozone assimilation, based on measurements between 1978 and today^19^, as described at www.bodekerscientific.com.
2. ozone values projected in the World Avoided scenario from 1974 to 2065^6^, which was calculated using the GEOS-CCM (Goddard Earth Observing System - Chemistry Climate model (NASA, USA)).
3. ozone values projected in the World Avoided scenario from 1974 to 2100^7^ which was calculated with the NIWA-UKCA (NIWA-UK Meteorological Office Climate Assessment) model.
4. ozone values projected for the World Expected scenario, as calculated with the NIWA-UKCA model. This scenario is the REF-C2 experiment described by Morgenstern *et al*.^20^. In this simulation, ozone-depleting substances follow the “WMO (2010) A1” scenario, which assumes compliance with the Montreal Protocol.

Differences between the two CCM models are summarized in Table 2. Details of both models are given elsewhere^16,20–24^.

**Table 2:** Comparison between the two CCM models used in this study.

Model	Horiz. resolution (lon × lat)	Vertical resolution	Full troposphere	Ocean Coupled?	End Date	Refs
GEOS-CCM	2.0° × 2.0°	55 levels to 0.01 hPa (80 km)	No	No	2065	^5, 23, 24^
NIWA-UKCA	3.75° × 2.5°	60 levels to 84 km	Yes	Yes	2100	^20– 22^

We note that future changes are dependent on the RCP scenario, and in particular depend on future changes in CO_2_, CH_4_, and N_2_O^25^. For example, a strong overshoot in ozone would be expected for RCP 8.5, and by the end of the 21^st^ century, differences between N_2_O and CH_4_ scenarios may account for differences in ozone larger than 5%.

For all scenarios, the clear-sky UVI at noon was interpolated from a 5D table of UVI as a function of ozone amount, solar zenith angle (SZA), altitude (pressure), aerosol optical depth, and surface albedo that had been pre-computed with the discrete-ordinate^26^ implementation in the TUV radiative transfer model^27^. Subsequent corrections were made to account for seasonal variations in Earth-Sun separation. In the World Avoided scenarios (Scenarios (2) and (3)) and World Expected scenario (Scenario (4)), the UVI was calculated for sea level with no aerosols and low surface albedo. Although both SO_2_ and NO_2_ can affect UVI, they have not been included in the clear-sky calculation. With the exception of San Diego, Thessaloniki, and Melbourne, sites considered in this paper are “clean-air” sites where the effect of absorption by these trace gases can be considered negligible.

To better approximate the measurements, corresponding UVI calculations using the ozone data at each site (Scenario (1)) were computed with TUV for the altitude at each site, assuming an aerosol optical depth of 0.05 (at wavelength 0.5 µm) with a single scattering albedo of 0.90 for all sites, and a representative (annually invariant) surface albedo, ranging from 0.05 at snow-free sites, to 0.98 at the South Pole.

As shown below, the comparison between the measured UVI data and the UVI data calculated from the assimilated ozone dataset (Scenario (1)) demonstrates that year-to-year variability in the UVI can be estimated with sufficient accuracy from ozone changes. The observed good agreement gives us confidence that UVI calculated from ozone for the period prior to the 1990s, when no direct UVI measurements are available, can be used to infer changes in UVI over this period. The comparisons with the two World Avoided scenarios were included to demonstrate the divergence between what has actually happened compared with what would have happened without the Montreal Protocol, using two independent model calculations.

## Results from Model Simulations

We begin by comparing projected ozone fields from the two World Avoided models (Scenarios (2) and (3)).

**Figure 1:**
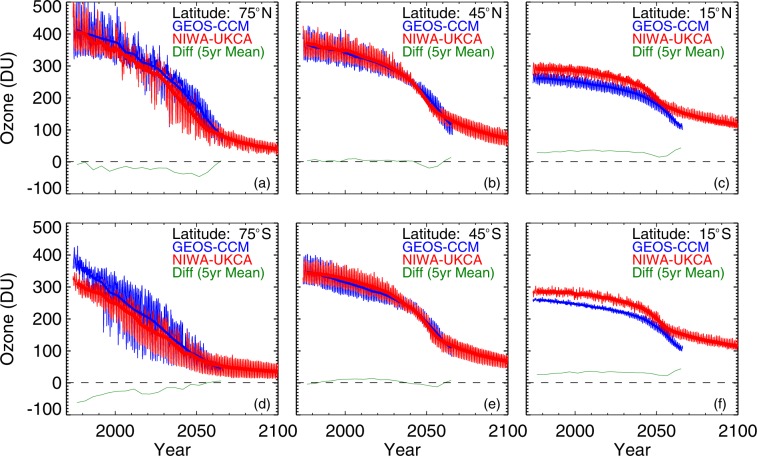
Comparison between projected ozone values in the GEOS-CCM (blue lines) and NIWA UKCA (red lines) World Avoided models for high (**a**,**d**), mid (**b**,**e**), and low (**c**,**f**) latitudes. Thicker lines are 5-year running means. Green lines indicate the smoothed difference between the two models (i.e., NIWA-UKCA minus GEOS-CCM). Plots for latitude 15° are representative of all equatorial latitudes, while those for 75° are representative for higher polar latitudes.

Predicted ozone values from these two models are compared for selected latitudes in Fig. 1. The two models are in general agreement in long-term behaviour, but also show significant differences over shorter time scales, especially at low latitudes. Close agreement is not expected at time scales of less than 5 years because there is a random component to the way modes of variability such as the quasi biennial oscillation (QBO) manifest themselves in the model runs. The systematic differences between the two models are within the usual range for chemistry-climate models^28^.

Modelled ozone data for Scenarios (2), (3) and (4) were compared with measured ozone data (Scenario (1)) and results are provided as Supplementary Data. In general, modelled and measured total ozone column amounts (TOCs) agree reasonably well. For the reference period 1978–1987 (i.e., the period between the year when ozone measurements from space became available globally and the year when the Montreal Protocol came into effect), TOCs calculated with the NIWA-UKCA model (both World Expected and World Avoided) for sites between 45°S and 45°N – which represents 71% of the globe –are on average 8 ± 4% (±1σ) higher than the measured TOCs. The maximum bias for this latitude range is 14%. TOCs calculated with the GEOS World Avoided model are slightly smaller. It overestimates the measured TOC by 2 ± 5% on average, with a maximum bias of 12%. Deviations between the modeled and measured TOCs become larger at higher latitudes, especially in the southern hemisphere. In this latitude range, differences can exceed 20% with biases from GEOS model exceeding those from the NIWA-UKCA model. These larger biases are partly a consequence of the low ozone amounts there and partly due to the difficulty to correctly model the destruction of ozone by heterogeneous chemical processes, which strongly depend on the temperature of the lower stratosphere. Furthermore, specific sites are not necessarily well represented by the zonal means used in the model. This is especially true for high-latitude southern hemisphere sites (i.e., Ushuaia and Palmer Station) in spring when the position of the ozone hole is frequently displaced toward the South American quadrant. Some of the discrepancy may also be due to interpolation errors in the ozone assimilation during the polar night, but this is irrelevant for changes in the UVI. Even though some uncertainties remain in the models, the agreement in geographic and seasonal patterns gives confidence that they can be used to project future changes. Finally, we also note that there may be errors in the model projections if factors such as changes in the Brewer-Dobson circulation are not properly parameterised.

Previous studies have also shown that the CCM models tend to overestimate ozone amounts. For example, the GEOS-CCM used previously^6^ has a high-ozone bias at mid to high latitudes^29^, which would lead to UVI estimates that are too low by approximately 10% at latitude 45° S. The green lines in Fig. 1 are the mean differences between the two models (smoothed), which shows that they depend on both latitude and time.

In the longer-term, projections by the two models are in good agreement. The reasonable level of consistency between these models adds further confidence to those predictions but also illustrates some significant differences.

It is interesting to note that by the year 2100, projected ozone declines are largest at high latitudes, and smallest in the tropics. By that time, the lowest mean ozone amounts would be in polar regions, and the highest in the tropics.

**Figure 2:**
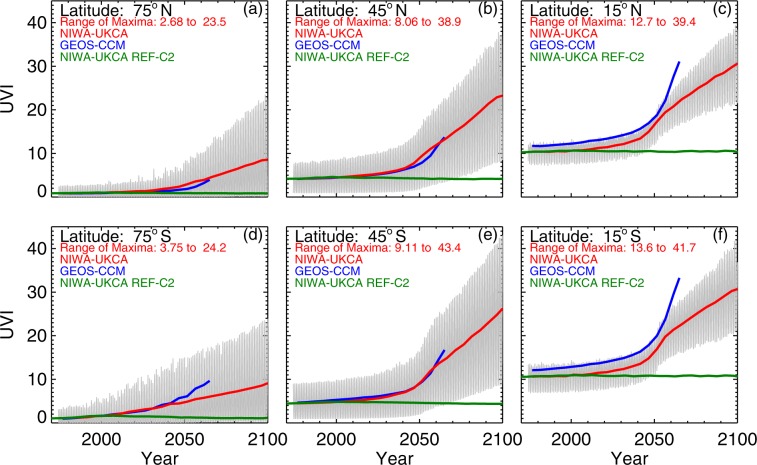
UVI calculated from World Avoided and World Expected simulations for the period 1960 to 2100 for high (**a**,**d**), mid (**b**,**e**), and low (**c**,**f**) latitudes. The shaded area shows the seasonal range of predictions calculated from the NIWA-UKCA World Avoided simulation. The upper envelope of the shaded range refers to peak values predicted for the summer and the lower envelope to minimum values predicted for winter. The red and blue lines indicate the 5-year running means for the NIWA-UKCA and GEOS-CCM World Avoided model simulations, respectively. The green line is the 5-year running mean of the NIWA-UKCA World Expected simulation, which assumes full compliance with the Montreal Protocol.

Corresponding changes in UVI from both World Avoided simulations, as well as from the World Expected simulation (Scenario (4)) are shown in Fig. 2. By 2100 the peak UVI at mid-latitudes would have increased a factor of four, and by much larger factors at higher latitudes. Even with the high ozone bias in the model, the calculated peak UVI would have exceeded 40 in over 70% of the area of the globe. Because of the latitudinal redistribution in ozone described above, at the end of the 21^st^ century, the largest clear-sky UVI values projected at mid-latitudes are similar to those in the tropics. This is in contrast to the present situation, where the peak UVI occurs in the tropics.

### Time series for lauder

**Figure 3:**
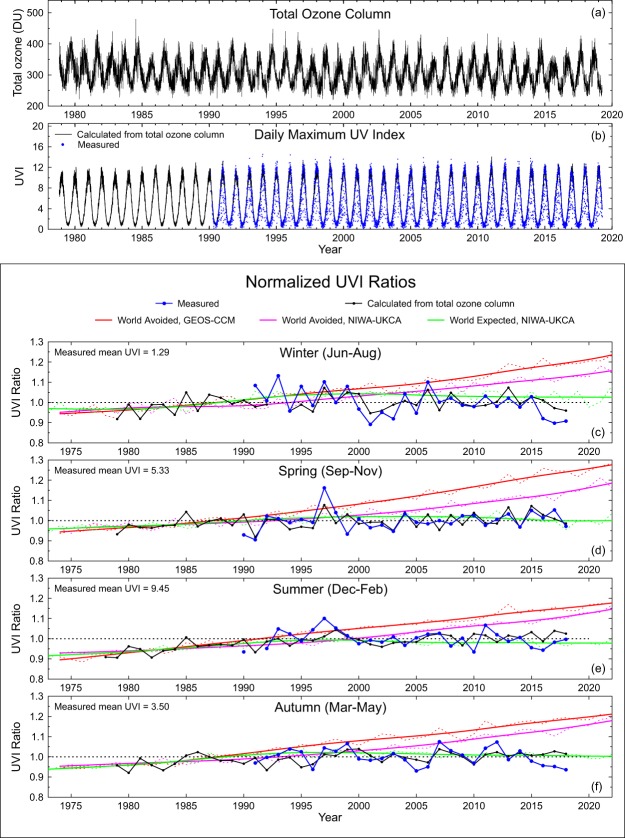
Total ozone column (**a**) and daily maximum UVI within 1 hour of local noon (**b**) at Lauder. Panels (**c**–**f**) show UVI changes for winter (**c**), spring (**d**), summer (**e**) and autumn (**f**) determined from measurements of the NDACC instruments (blue), calculated from total ozone column (black), and projected by the two World Avoided (red and magenta) and World Expected (green) CCM model runs. UVI changes were normalized as described in the text and the “UVI ratios” shown in Panels (c–f) are ratios relative to these normalizations. Note that for the season that spans two years (summer at this southern hemisphere site), the year label refers to the year at the start of the season. For example, the summer of Dec 2017 to Feb 2018 is plotted at 2017.

We now compare these UVI projections from the CCM models with observed changes in UVI and with those expected from clear-sky calculations using the assimilated ozone values. Results for Lauder are shown in Fig. 3. Figure 3a displays the time series of daily ozone derived from the NIWA/BS ozone assimilation for the period since satellite measurements became available. In Fig. 3b, the peak UVI measured within one hour of local noon is compared with the UVI calculated for clear-sky conditions using the NIWA/BS ozone data. There is good agreement in the seasonal range between measured and modelled UVI.

Because of the large seasonal and day-to-day variability, it is difficult to see any long-term effects (other than in the upper envelope of data) in Fig. 3b. The lower four panels of Fig. 3 redress this by considering seasonal means, with each data point representing the mean over different 3-month periods. Because of the previously discussed biases in calculated and modelled ozone, all data shown in Fig. 3c–f were normalized. The measured UVI values were normalized to the average of all values, while the calculated UVIs were normalized to the average of only those years with measurements. The normalized peak UVI changes derived from measurements (blue lines) are compared with those calculated from the assimilated ozone dataset (black lines). Also shown in these panels are the corresponding UVI changes that would have occurred according to the World Avoided calculations reported by the GEOS-CCM model (broken red lines) and the NIWA-UKCA model (broken magenta lines). UVI values in the World Expected are also shown (broken green lines). The heavier solid lines for each model run include smoothing with an approximating spline. Both World Avoided datasets and the World Expected dataset were normalized to the average of the calculated UVI values of the years 1978 to 1987; the decade that immediately preceded the signing of the Montreal Protocol. Corresponding plots for all other sites are in Supplementary Data.

In the period prior to 2020 shown in Fig. 3, projected changes are generally smaller with the NIWA-UKCA model than with the GEOS-CCM model. However, this is not everywhere the case. At northern mid-latitudes, the two models are in close agreement (see Supplementary Data). They are also similar at high southern latitudes during summer, while the NIWA-UKCA values are larger at high northern latitudes. Reasons for these model differences are outside the scope of the present study.

As expected, the World Expected line (green) is usually below the World Avoided line (pink). However, for a short period in the late 1990s, which is most pronounced in the winter, it is higher. Differences of this magnitude are within the range of spread between model runs of about 5%.

### UVI in summer

**Figure 4:**
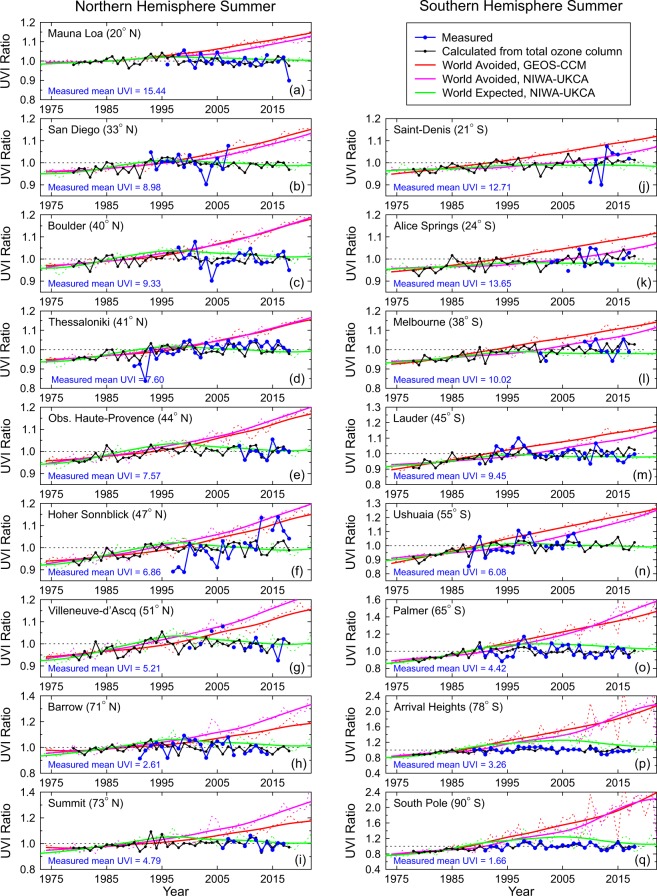
Comparison of normalized UVI ratios for summer months at all sites between measurements, calculations for clear skies based on the ozone assimilation, and clear-sky models for the World Avoided and World Expected scenarios. Note that the vertical axis scale varies between panels.

Possible changes in UV radiation during summer are most relevant from an environmental and health perspective. In Fig. 4, we compare measured, calculated, and projected UVIs for summer at all 17 sites in Table 1. The figure shows that the World Avoided trends depend greatly on latitude and that differences between the two model runs are also latitude-dependent. For example, at latitude 40°N, there is close agreement between the two models (for other seasons, see Supplementary Data).

With the exception of Hoher Sonnblick, the measured data from all sites closely match the calculated values and are much closer to the World Expected curves than the World Avoided curves. However, there is a significant divergence between the measured values and World Expected values at higher latitudes: measured and calculated UVI ratios are more or less constant over time while the World Expected curves have a broad maximum during the first decade of the 21^st^ century (last two panels of Fig. 4). This suggests that the impact of the large spring-time ozone depletions persists for too long into the following summer in the model. This is a well-known limitation of CCMs^28^ for which the causes are not fully understood, and is an area of current research.

The measurements at Hoher Sonnblick (Panel f) show a continuation of the previously reported upward trend in summertime UVI^30^, which was attributed to changes in aerosols or cloud cover. This increase is not present in all seasons, so it is not a calibration artefact. However, there are several seasonal gaps in the data, and the year-to-year variability is also much greater at this mountainous site (see Supplementary Data), possibly due to variable snow cover.

At some other sites, the length of the time series is too short for reliable trend estimates. Only nine of the 17 sites have more than 20 years of data coverage.

Figure 4 also indicates that UVI ratios calculated from the assimilated ozone data are generally less than unity prior to the 1990s and before the start of UV measurements. The slope in the calculated data is generally consistent with the slope in the datasets of the three CCM models. These model calculations imply that considerable changes in summer UVI occurred between 1978 and 1990 (about 5% at northern mid-latitude sites, up to 10% at southern mid-latitude sites and up to 20% at the three Antarctic sites). However, direct UVI observations needed to confirm these changes do not exist.

After accounting for differences in elevation between the sites, we note that latitude-for-latitude, the mean summer UVI values tend to be larger at the southern hemisphere (SH) sites compared with the northern hemisphere (NH) sites, as has been predicted and observed previously^31–34^. This NH/SH difference is as expected. For example, Fig. 2 shows that at 45°S, the peak UVI value is larger than at 45°N by approximately 13%. Approximately half of this difference is due to ozone differences and half is due to Sun-Earth separation differences. The much larger NH/SH differences reported in the past^31–34^ also include effects of clouds and aerosols, which were not considered in the CCM calculations. The NH/SH asymmetry is projected to be only slightly smaller by year 2100.

### UVI in spring at high latitudes

**Figure 5:**
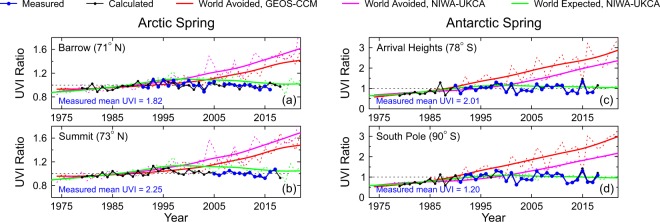
Comparison of UVI in spring at polar sites between measurements, calculations for clear skies based on the ozone assimilation, and clear-sky models for the World Avoided and World Expected scenarios.

In Fig. 5, we compare normalized measured, calculated, World Avoided, and World Expected UVIs in spring for the Arctic and Antarctic sites included in this study, where the largest long-term changes in UVI have been observed. Note the expanded vertical axis scale compared with that in Fig. 4. For these high-latitude sites, the “calculated” and “measured” data sets are in almost perfect agreement, particularly in Antarctica, indicating that ozone variation is the largest contributor to UVI changes. Variability of clouds and albedo is a minor contributor in comparison.

The “hump” in the World Expected data noted previously for the summer data (Fig. 4), which peaks in the early part of the 21^st^ century, is also present in the spring data (Fig. 5). However, in the spring period, there is also a hint of this in the measured and calculated data for the two Antarctic sites and perhaps also at Summit. Data from Barrow show more variability, as one would expect from an Arctic coastal site that is affected by changes in albedo from snow and sea ice.

### Decadal trends in UVI

Due to the less complete data coverage early in the period, our statistical analysis is restricted to the period since 1996. This period also excludes effects from the eruption of Mt Pinatubo, which led to significant effects on ozone and UV at some locations^35–38^. The period since 1996 also includes approximately two complete 11-year solar cycles, which cause variations in ozone of ±2%^39,40^ that would lead in turn to a modulation of similar relative magnitude, but of opposite sign, in the UVI.

Even for the period since 1996, measured data are unfortunately incomplete at some sites, and only a small number of sites have full data coverage through to the end of 2018 (see Table 1). For example, due to funding restrictions, measurements were terminated in 2017 at Arctic sites that were initiated by Biospherical Instruments (BSI). Their time series from Ushuaia and San Diego are also restricted to the period before 2010 and 2008 respectively. There was also a gap from 2003 to 2008 in Melbourne data. Of the 17 sites, only three (Lauder, Palmer, and Thessaloniki) have near-complete data coverage for the full period from 1996 to 2018. However, six others (Barrow, Hoher Sonnblick, Boulder, Mauna Loa, Arrival Heights and South Pole) have only a few years of missing data over that period. Greatest confidence should be placed on data from these nine stations.

**Figure 6:**
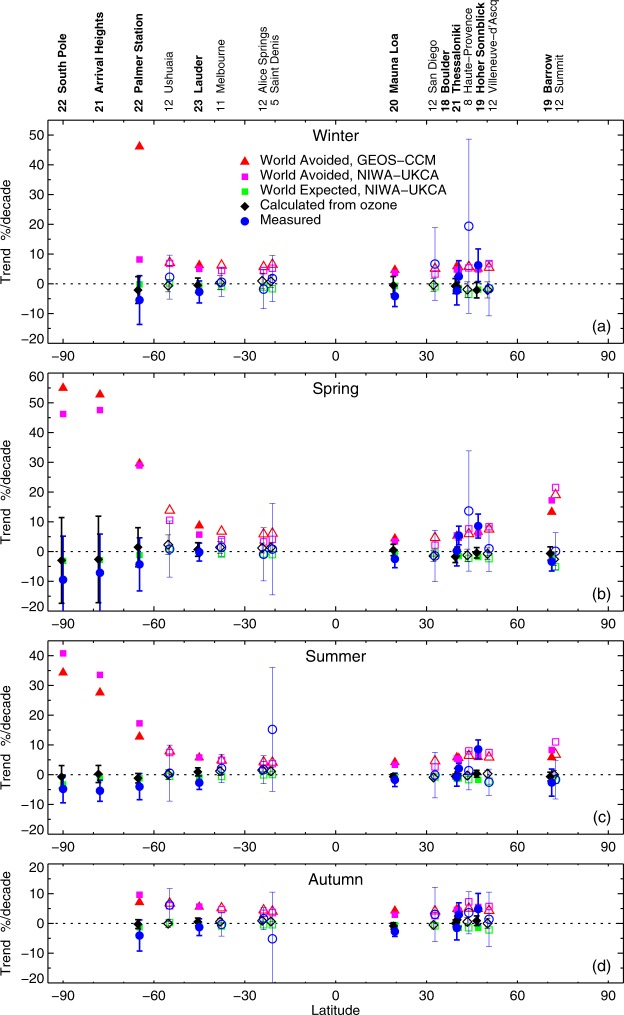
Calculated decadal trends in measured UVI since 1996 (or from the data start year, if later than 1996) as a function of site latitude, compared with those calculated for clear skies from observed ozone, and as calculated by the two World Avoided model runs and the World Expected run for each season (**a**–**d**). Sites where the time series spans 20 years or more are denoted by bold text and solid symbols. The number of years of data included in the trend analysis at each site is indicated beside the site name. If data from some seasons are missing, this number can be less than the total number of years. Error bars shown are 2-σ uncertainties of the regression model.

There are other possible forcing mechanisms that drive interannual variability in UVI. These include variability due to the QBO or the *El Nino* Southern Oscillation (ENSO). However, over time scales as long as 22 years, any such effects are small, and have not been considered in the statistical analysis. In these seasonal averages, where each data point is separated from the previous value by a period of 9 months, any statistical auto-correlation effects should also be small. In the trend analysis, we have assumed that any long-term changes over the period 1996 to 2018 could be represented by a linear change, which is a reasonable assumption (e.g., see Fig. 4). Results of the statistical analysis are shown in Fig. 6. The results are not particularly sensitive to the start year, resulting in a similar picture (not shown) if the start-date for the analysis is changed by 1 or 2 years.

Note that the error bars shown for the trend estimates in Fig. 6 are 2-σ uncertainties of the regression model, which also include small random uncertainties in the measurements that propagate to the 90-day averages analysed here. However, systematic errors due to potential long-term drifts in the measurements (e.g., due to the transfer of irradiance scales between calibration standards) are not included.

Figure 6 shows the following:

1. *The two World Avoided Simulations give similar trends:* With the exception of Palmer Station in winter, there is good agreement between the trends derived from the two World Avoided simulations. (At Palmer Station, the sun is only 2° above the horizon (SZA_min_ = 88°) at the winter solstice, so peak UVI values during this period are small.) The calculated UVI is also sensitive to small modelling and sampling differences (see Supplementary Data). Also, as shown in Fig. 1 (lower left panel), there are significant differences in the seasonal variability between the two model projections in this latitude region and period.
2. *UV Trends are large in the World Avoided Simulations:* In the World Avoided (without the Montreal Protocol), UVI levels would have increased over the last 22 years by approximately 50% per decade at high southern latitudes in spring, and by 30% per decade in summer. Increases of up to 20% per decade would have been seen at northern high latitudes. At midlatitudes, the increases would have been approximately 5–10% per decade. Such changes would have been clearly detectable in the measurement data.
3. *Measurements Differ from the World Avoided Simulations:* In the spring and summer, the observed trends for all nine sites with good data coverage (solid symbols in Fig. 6) are significantly different from the World Avoided trends at all mid- and high-latitudes in the southern hemisphere, and at high latitudes in the northern hemisphere. At northern mid-latitudes, differences between measured trends and trends from the World Avoided simulations are significant in summer at all sites except Hoher Sonnblick, but in other seasons the results are more mixed.
4. *Measurements follow the World Expected Simulations:* For all sites, trends calculated from the measurements are consistent with the World Expected scenario, though uncertainties are large at Hoher Sonnblick, and other sites (e.g., San Diego, Saint Denis and Obs. Haute-Provence), where there are only a few years of observation. While variability in UVI is close to that predicted by changes in ozone at the southern hemisphere sites, the situation is more complex at northern mid-latitude sites, such as Hoher Sonnblick, where the effects of changes in aerosols and clouds are more important in some seasons.
5. *Evidence of a Downward Tendency in UVI since 1996 in the Southern Hemisphere:* At the three Antarctic sites (South Pole, Arrival Heights, and Palmer Station) the measured UVIs reveal decreases of about 5% per decade during summer, however, only the trend at Arrival Heights is significant at the 95% confidence level. We note that UVIs calculated from changes in ozone show almost no change, suggesting that changes in albedo or cloud cover contribute to the trends in the measured UVIs. We also note that measurements at these three remote sites are potentially affected by long-term drifts of approximately 1% per decade, which are associated with hardware modifications and changes in calibration standards. Even if these drifts were confirmed, the downward trend at Arrival Heights would remain statistically significant. At the South Pole and Arrival Heights, both measured and calculated UVIs appear to have decreased during spring (the period most affected by the ozone hole), however, trends calculated from these changes are not yet statistically significant. At Lauder, small downward trends in measured UVI are observed for summer and autumn, which are on the verge of being statistically significant. These trends are generally consistent with trends derived from the NIWA/BS ozone dataset, although throughout the southern hemisphere, trends are systematically more negative for measurements than for calculations for all seasons.

## Conclusions

Where data are available in the early 1990s, most sites show evidence of increasing UVI in the earliest part of the record when ozone was declining. However, the short length of the data record prior to 2000, and possible interference from the eruption of Mt Pinatubo, hinder our ability to ascribe changes in UVI to changes in ozone.

In most cases, we find that calculated and measured trends have been small and are in most cases not significantly different from zero. With the exception of one site (Hoher Sonnblick), they also agree with each other to within their error bars, and with trends calculated with the World Expected model.

At clean-air sites in the southern hemisphere, the UVI has followed the World Expected scenario within the limits of the measurement uncertainty. Differences between measurements and the two World Avoided models are already highly significant in the Arctic, and at southern hemisphere sites, especially in Antarctica. The situation is more complex at mid-latitudes in the northern hemisphere, where the effects of changes in aerosol and clouds mask effects of ozone.

For winter and autumn, changes observed at mid-latitude sites are mixed. Measurements show a tendency towards decreasing UVI in the southern hemisphere and increasing UVI in the northern hemisphere. However, few of the observed long-term changes since 1996 are statistically significant.

A statistically significant decrease in measured summertime UVI has been observed at the Antarctic site Arrival Heights, and the reduction in UVI at Lauder is close to being statistically significant at the 95% level. However, at both sites, trends calculated from ozone remain close to zero, showing that the reductions in UVI must be due to other factors (e.g., changes in cloud, aerosol, or surface albedo). Measurements during the last few years at Lauder also show signs of decreases in UVI, which also cannot be attributed to ozone changes.

The Montreal Protocol has been effective in curbing increases in harmful UVI. Without the Montreal Protocol, UVI values at northern and southern latitudes <50° would by now be 10 to 20% larger in all seasons compared to UVIs observed during the early 1990s. These changes would have had grave consequences for public health and would have led to increases in skin cancer occurrences^41^. For latitudes >50°S, UVI values would have increased over the same period between 25% (Ushuaia in summer and autumn) to more than 100% (South Pole in spring and summer).

UVI values in the future remain uncertain because of:

- possible volcanic eruptions, which could temporarily exacerbate ozone depletion as long as chlorine levels remain elevated;
- interactions with other aspects of climate change, such as changes in clouds and aerosols;
- slowly varying natural modes of variability^42^, such as the Interdecadal Pacific Oscillation^43^, which may affect cloud cover but are not included in the study;
- effects of increasing greenhouse gases, which will lead to stratospheric cooling and changes in dynamics that may subsequently cause ozone to increase above levels observed in 1980 outside polar regions;
- non-compliance to the Montreal Protocol, such as found in a recent study^44^.

The reduced number of operational sites measuring UV irradiance is concerning. The value of time series data increases with the length of the record, as shown by the smaller error bars for the longer-term measurement sites in Fig. 6. Unfortunately, there are only a few high-quality NDACC sites with data from the early 1990s that are still operational. It is important that they continue to be supported in case of unexpected future changes.

## Supplementary information

Success of Montreal Protocol Demonstrated by Comparing High-Quality UV Measurements with “World Avoided” Calculations from Two Chemistry-Climate Models
