# Supplementary material for: Success of Montreal Protocol Demonstrated by Comparing High-Quality UV Measurements with “World Avoided” Calculations from Two Chemistry-Climate Models
Source: Sci Rep. 2019 Sep 3;9:12332. doi: 10.1038/s41598-019-48625-z (PMC6722083; doi:10.1038/s41598-019-48625-z)
Supplement: Supplementary file 1 — Success of Montreal Protocol Demonstrated by Comparing High-Quality UV Measurements with “World Avoided” Calculations from Two Chemistry-Climate Models [file 41598_2019_48625_MOESM1_ESM.pdf]

Success of Montreal Protocol Demonstrated by Comparing High-Quality UV Measurements with "World Avoided" Calculations from Two Chemistry-Climate Models

Richard McKenzie, Germar Bernhard, Ben Liley, Patrick Disterhoft, Steve Rhodes, Alkiviadis Bais, Olaf Morgenstern, Paul Newman, Luke Oman, Colette Brogniez, Stana Simic

Supplementary Data

The supplementary data consists of:

Page	Content
2 – 5	Four multi-panel figures comparing measured (Scenario (1)) and projected (Scenarios (2) – (4)) long-term changes in total ozone columns (TOCs) at each of the 17 sites discussed in the paper. The 4 pages show results for each season (winter, spring, summer, autumn). Numbers shown on each panel indicate for each scenario the average TOC in Dobson Units, calculated over the 10-year reference period 1978-1987. The percentage difference between the average TOCs of the three models (NIWA-UKCA, World Expected; NIWA-UKCA, World Avoided; and GEOS-CCM, World avoided) and the average of the measured TOCs are also indicated.
6 – 22	17 multi-panel plots showing time series for ozone and UVI changes, similar to Figure 3 in the paper. Each of the 17 pages shows results for a different site.
23 – 26	Four multi-panel plots comparing measured and projected long term changes in UVI at each of the 17 sites, similar to Figure 4 in the paper. The four pages show results for each season (winter, spring, summer, autumn).

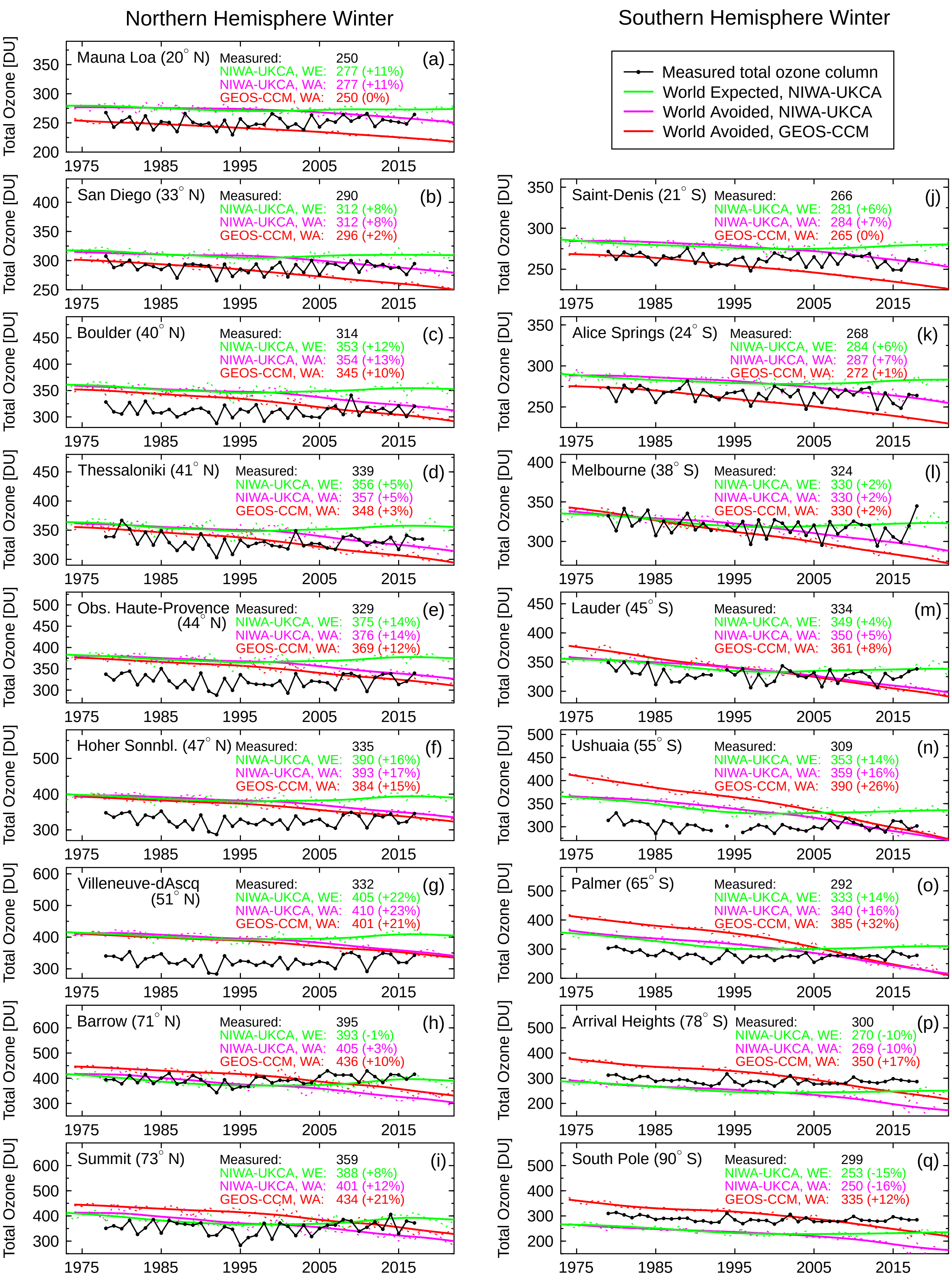

Northern Hemisphere Winter
Southern Hemisphere Winter
Measured total ozone column
World Expected, NIWA-UKCA
World Avoided, NIWA-UKCA
World Avoided, GEOS-CCM
Total Ozone [DU]
1975 1985 1995 2005 2015
Mauna Loa (20° N) (a)
Measured: 250
NIWA-UKCA, WE: 277 (+11%)
NIWA-UKCA, WA: 277 (+11%)
GEOS-CCM, WA: 250 (0%)
San Diego (33° N) (b)
Measured: 290
NIWA-UKCA, WE: 312 (+8%)
NIWA-UKCA, WA: 312 (+8%)
GEOS-CCM, WA: 296 (+2%)
Boulder (40° N) (c)
Measured: 314
NIWA-UKCA, WE: 353 (+12%)
NIWA-UKCA, WA: 354 (+13%)
GEOS-CCM, WA: 345 (+10%)
Thessaloniki (41° N) (d)
Measured: 339
NIWA-UKCA, WE: 356 (+5%)
NIWA-UKCA, WA: 357 (+5%)
GEOS-CCM, WA: 348 (+3%)
Obs. Haute-Provence (44° N) (e)
Measured: 329
NIWA-UKCA, WE: 375 (+14%)
NIWA-UKCA, WA: 376 (+14%)
GEOS-CCM, WA: 369 (+12%)
Hoher Sonnbl. (47° N) (f)
Measured: 335
NIWA-UKCA, WE: 390 (+16%)
NIWA-UKCA, WA: 393 (+17%)
GEOS-CCM, WA: 384 (+15%)
Villeneuve-dAscq (51° N) (g)
Measured: 332
NIWA-UKCA, WE: 405 (+22%)
NIWA-UKCA, WA: 410 (+23%)
GEOS-CCM, WA: 401 (+21%)
Barrow (71° N) (h)
Measured: 395
NIWA-UKCA, WE: 393 (-1%)
NIWA-UKCA, WA: 405 (+3%)
GEOS-CCM, WA: 436 (+10%)
Summit (73° N) (i)
Measured: 359
NIWA-UKCA, WE: 388 (+8%)
NIWA-UKCA, WA: 401 (+12%)
GEOS-CCM, WA: 434 (+21%)
Saint-Denis (21° S) (j)
Measured: 266
NIWA-UKCA, WE: 281 (+6%)
NIWA-UKCA, WA: 284 (+7%)
GEOS-CCM, WA: 265 (0%)
Alice Springs (24° S) (k)
Measured: 268
NIWA-UKCA, WE: 284 (+6%)
NIWA-UKCA, WA: 287 (+7%)
GEOS-CCM, WA: 272 (+1%)
Melbourne (38° S) (l)
Measured: 324
NIWA-UKCA, WE: 330 (+2%)
NIWA-UKCA, WA: 330 (+2%)
GEOS-CCM, WA: 330 (+2%)
Lauder (45° S) (m)
Measured: 334
NIWA-UKCA, WE: 349 (+4%)
NIWA-UKCA, WA: 350 (+5%)
GEOS-CCM, WA: 361 (+8%)
Ushuaia (55° S) (n)
Measured: 309
NIWA-UKCA, WE: 353 (+14%)
NIWA-UKCA, WA: 359 (+16%)
GEOS-CCM, WA: 390 (+26%)
Palmer (65° S) (o)
Measured: 292
NIWA-UKCA, WE: 333 (+14%)
NIWA-UKCA, WA: 340 (+16%)
GEOS-CCM, WA: 385 (+32%)
Arrival Heights (78° S) (p)
Measured: 300
NIWA-UKCA, WE: 270 (-10%)
NIWA-UKCA, WA: 269 (-10%)
GEOS-CCM, WA: 350 (+17%)
South Pole (90° S) (q)
Measured: 299
NIWA-UKCA, WE: 253 (-15%)
NIWA-UKCA, WA: 250 (-16%)
GEOS-CCM, WA: 335 (+12%)

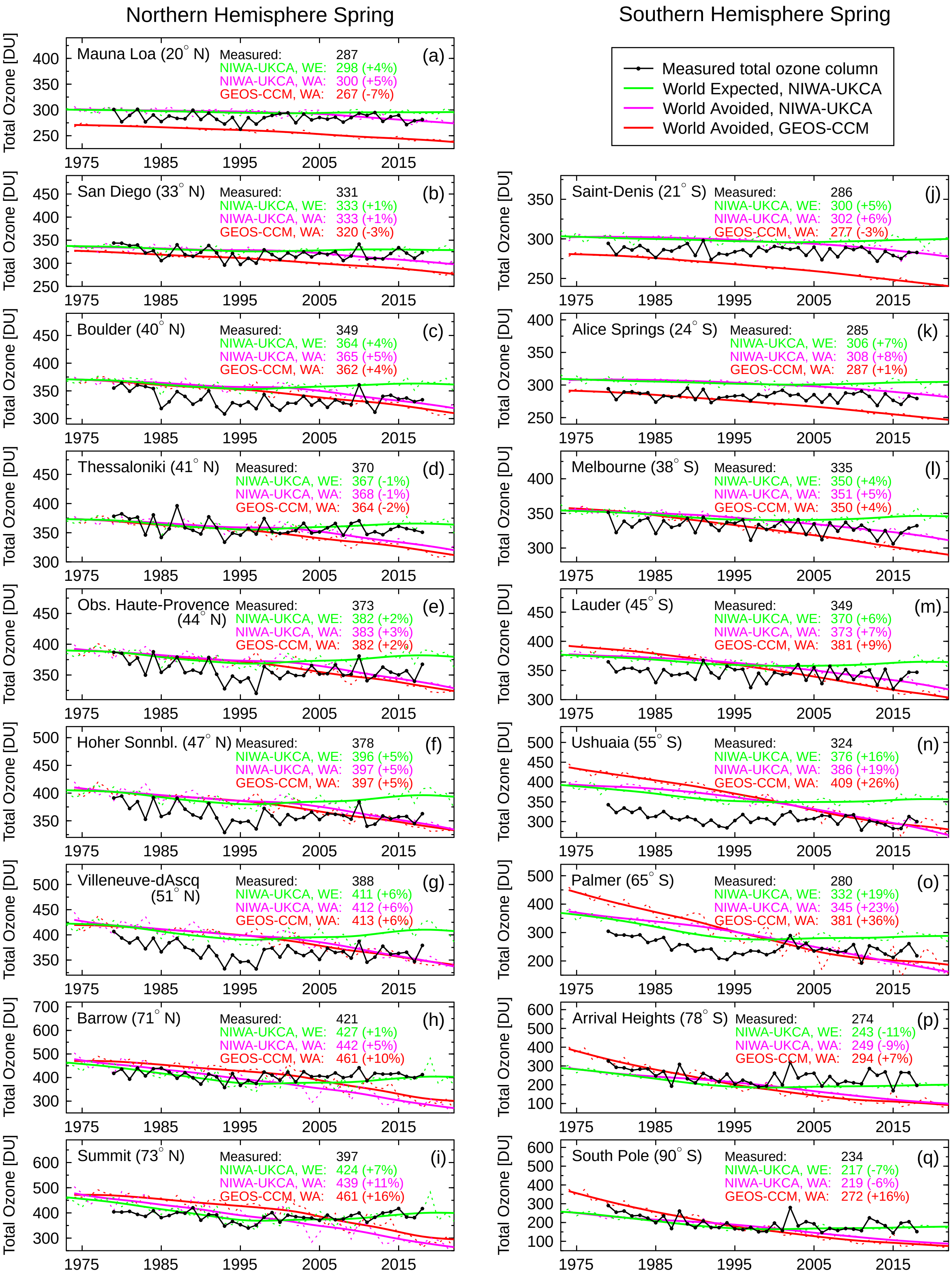

Northern Hemisphere Spring
Southern Hemisphere Spring
Measured total ozone column
World Expected, NIWA-UKCA
World Avoided, NIWA-UKCA
World Avoided, GEOS-CCM
Total Ozone [DU]
Mauna Loa (20° N) Measured: 287 NIWA-UKCA, WE: 298 (+4%) NIWA-UKCA, WA: 300 (+5%) GEOS-CCM, WA: 267 (-7%) (a)
San Diego (33° N) Measured: 331 NIWA-UKCA, WE: 333 (+1%) NIWA-UKCA, WA: 333 (+1%) GEOS-CCM, WA: 320 (-3%) (b)
Boulder (40° N) Measured: 349 NIWA-UKCA, WE: 364 (+4%) NIWA-UKCA, WA: 365 (+5%) GEOS-CCM, WA: 362 (+4%) (c)
Thessaloniki (41° N) Measured: 370 NIWA-UKCA, WE: 367 (-1%) NIWA-UKCA, WA: 368 (-1%) GEOS-CCM, WA: 364 (-2%) (d)
Obs. Haute-Provence (44° N) Measured: 373 NIWA-UKCA, WE: 382 (+2%) NIWA-UKCA, WA: 383 (+3%) GEOS-CCM, WA: 382 (+2%) (e)
Hoher Sonnbl. (47° N) Measured: 378 NIWA-UKCA, WE: 396 (+5%) NIWA-UKCA, WA: 397 (+5%) GEOS-CCM, WA: 397 (+5%) (f)
Villeneuve-dAscq (51° N) Measured: 388 NIWA-UKCA, WE: 411 (+6%) NIWA-UKCA, WA: 412 (+6%) GEOS-CCM, WA: 413 (+6%) (g)
Barrow (71° N) Measured: 421 NIWA-UKCA, WE: 427 (+1%) NIWA-UKCA, WA: 442 (+5%) GEOS-CCM, WA: 461 (+10%) (h)
Summit (73° N) Measured: 397 NIWA-UKCA, WE: 424 (+7%) NIWA-UKCA, WA: 439 (+11%) GEOS-CCM, WA: 461 (+16%) (i)
Saint-Denis (21° S) Measured: 286 NIWA-UKCA, WE: 300 (+5%) NIWA-UKCA, WA: 302 (+6%) GEOS-CCM, WA: 277 (-3%) (j)
Alice Springs (24° S) Measured: 285 NIWA-UKCA, WE: 306 (+7%) NIWA-UKCA, WA: 308 (+8%) GEOS-CCM, WA: 287 (+1%) (k)
Melbourne (38° S) Measured: 335 NIWA-UKCA, WE: 350 (+4%) NIWA-UKCA, WA: 351 (+5%) GEOS-CCM, WA: 350 (+4%) (l)
Lauder (45° S) Measured: 349 NIWA-UKCA, WE: 370 (+6%) NIWA-UKCA, WA: 373 (+7%) GEOS-CCM, WA: 381 (+9%) (m)
Ushuaia (55° S) Measured: 324 NIWA-UKCA, WE: 376 (+16%) NIWA-UKCA, WA: 386 (+19%) GEOS-CCM, WA: 409 (+26%) (n)
Palmer (65° S) Measured: 280 NIWA-UKCA, WE: 332 (+19%) NIWA-UKCA, WA: 345 (+23%) GEOS-CCM, WA: 381 (+36%) (o)
Arrival Heights (78° S) Measured: 274 NIWA-UKCA, WE: 243 (-11%) NIWA-UKCA, WA: 249 (-9%) GEOS-CCM, WA: 294 (+7%) (p)
South Pole (90° S) Measured: 234 NIWA-UKCA, WE: 217 (-7%) NIWA-UKCA, WA: 219 (-6%) GEOS-CCM, WA: 272 (+16%) (q)
1975 1985 1995 2005 2015

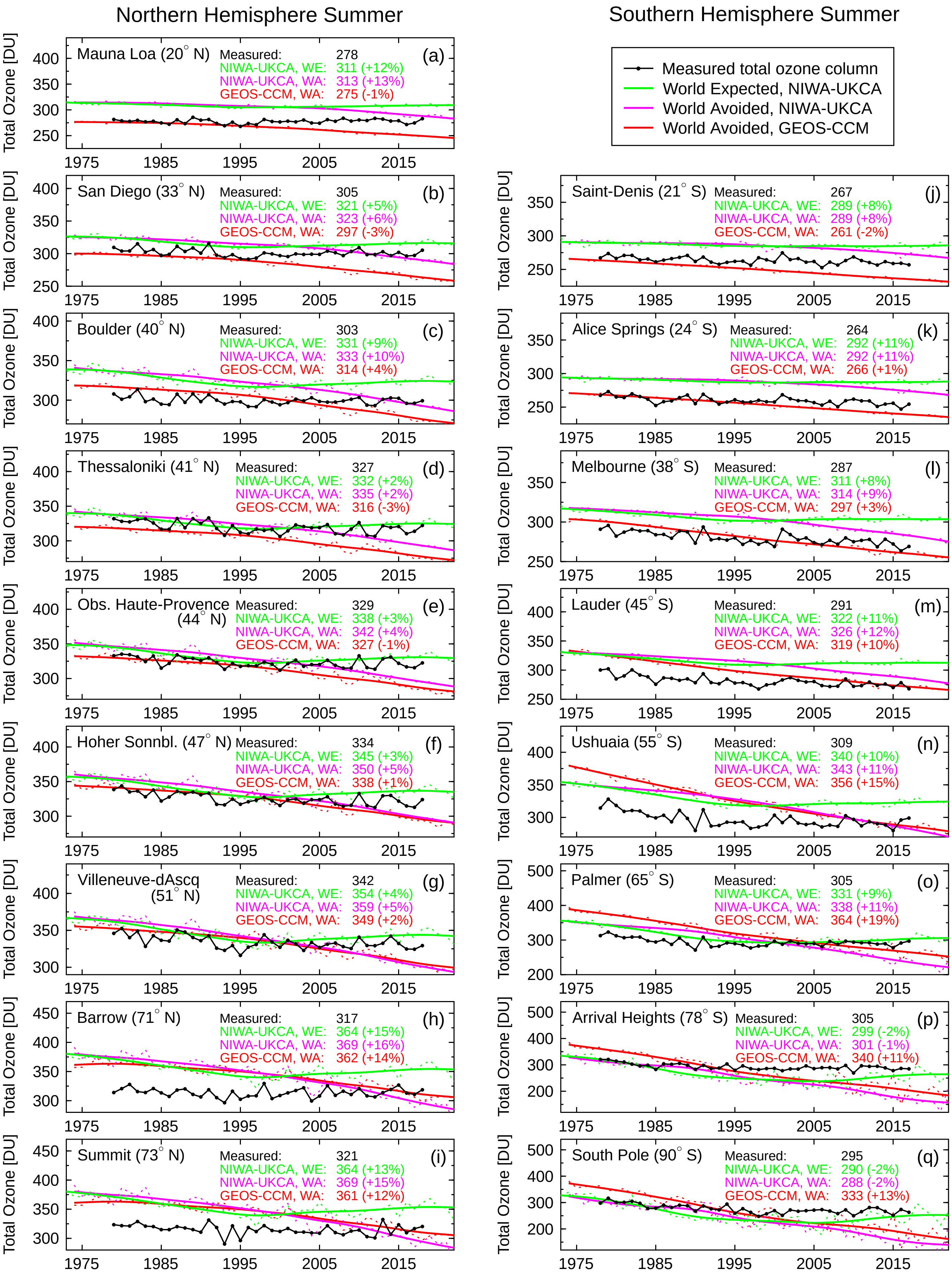
Northern Hemisphere Summer
Southern Hemisphere Summer
Measured total ozone column
World Expected, NIWA-UKCA
World Avoided, NIWA-UKCA
World Avoided, GEOS-CCM
Total Ozone [DU]
Mauna Loa (20° N) Measured: 278 NIWA-UKCA, WE: 311 (+12%) NIWA-UKCA, WA: 313 (+13%) GEOS-CCM, WA: 275 (-1%) (a)
San Diego (33° N) Measured: 305 NIWA-UKCA, WE: 321 (+5%) NIWA-UKCA, WA: 323 (+6%) GEOS-CCM, WA: 297 (-3%) (b)
Boulder (40° N) Measured: 303 NIWA-UKCA, WE: 331 (+9%) NIWA-UKCA, WA: 333 (+10%) GEOS-CCM, WA: 314 (+4%) (c)
Thessaloniki (41° N) Measured: 327 NIWA-UKCA, WE: 332 (+2%) NIWA-UKCA, WA: 335 (+2%) GEOS-CCM, WA: 316 (-3%) (d)
Obs. Haute-Provence (44° N) Measured: 329 NIWA-UKCA, WE: 338 (+3%) NIWA-UKCA, WA: 342 (+4%) GEOS-CCM, WA: 327 (-1%) (e)
Hoher Sonnbl. (47° N) Measured: 334 NIWA-UKCA, WE: 345 (+3%) NIWA-UKCA, WA: 350 (+5%) GEOS-CCM, WA: 338 (+1%) (f)
Villeneuve-dAscq (51° N) Measured: 342 NIWA-UKCA, WE: 354 (+4%) NIWA-UKCA, WA: 359 (+5%) GEOS-CCM, WA: 349 (+2%) (g)
Barrow (71° N) Measured: 317 NIWA-UKCA, WE: 364 (+15%) NIWA-UKCA, WA: 369 (+16%) GEOS-CCM, WA: 362 (+14%) (h)
Summit (73° N) Measured: 321 NIWA-UKCA, WE: 364 (+13%) NIWA-UKCA, WA: 369 (+15%) GEOS-CCM, WA: 361 (+12%) (i)
Saint-Denis (21° S) Measured: 267 NIWA-UKCA, WE: 289 (+8%) NIWA-UKCA, WA: 289 (+8%) GEOS-CCM, WA: 261 (-2%) (j)
Alice Springs (24° S) Measured: 264 NIWA-UKCA, WE: 292 (+11%) NIWA-UKCA, WA: 292 (+11%) GEOS-CCM, WA: 266 (+1%) (k)
Melbourne (38° S) Measured: 287 NIWA-UKCA, WE: 311 (+8%) NIWA-UKCA, WA: 314 (+9%) GEOS-CCM, WA: 297 (+3%) (l)
Lauder (45° S) Measured: 291 NIWA-UKCA, WE: 322 (+11%) NIWA-UKCA, WA: 326 (+12%) GEOS-CCM, WA: 319 (+10%) (m)
Ushuaia (55° S) Measured: 309 NIWA-UKCA, WE: 340 (+10%) NIWA-UKCA, WA: 343 (+11%) GEOS-CCM, WA: 356 (+15%) (n)
Palmer (65° S) Measured: 305 NIWA-UKCA, WE: 331 (+9%) NIWA-UKCA, WA: 338 (+11%) GEOS-CCM, WA: 364 (+19%) (o)
Arrival Heights (78° S) Measured: 305 NIWA-UKCA, WE: 299 (-2%) NIWA-UKCA, WA: 301 (-1%) GEOS-CCM, WA: 340 (+11%) (p)
South Pole (90° S) Measured: 295 NIWA-UKCA, WE: 290 (-2%) NIWA-UKCA, WA: 288 (-2%) GEOS-CCM, WA: 333 (+13%) (q)
1975 1985 1995 2005 2015

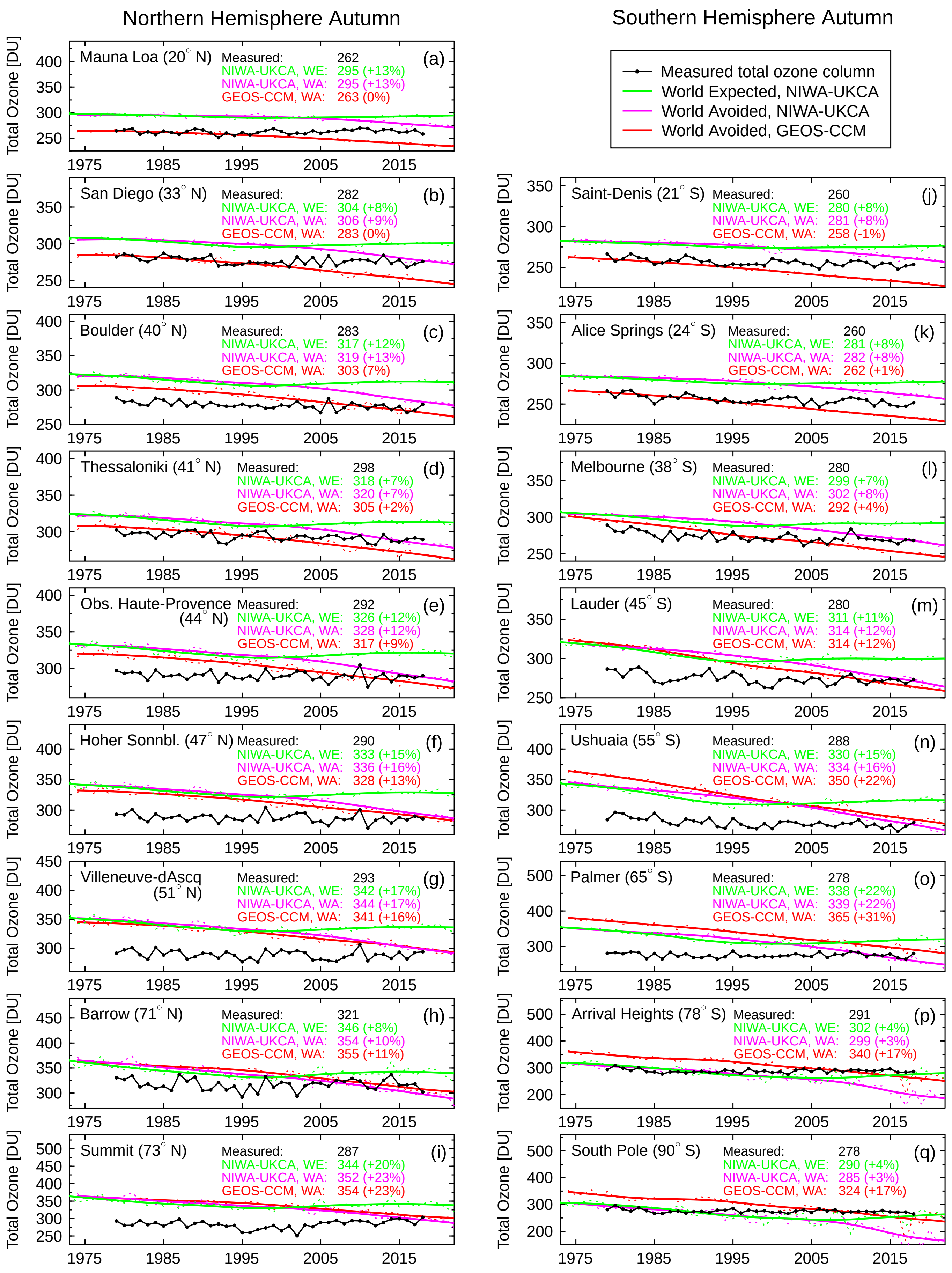
Northern Hemisphere Autumn
Southern Hemisphere Autumn
Measured total ozone column
World Expected, NIWA-UKCA
World Avoided, NIWA-UKCA
World Avoided, GEOS-CCM
Total Ozone [DU]
Mauna Loa (20° N) Measured: 262 NIWA-UKCA, WE: 295 (+13%) NIWA-UKCA, WA: 295 (+13%) GEOS-CCM, WA: 263 (0%) (a)
San Diego (33° N) Measured: 282 NIWA-UKCA, WE: 304 (+8%) NIWA-UKCA, WA: 306 (+9%) GEOS-CCM, WA: 283 (0%) (b)
Boulder (40° N) Measured: 283 NIWA-UKCA, WE: 317 (+12%) NIWA-UKCA, WA: 319 (+13%) GEOS-CCM, WA: 303 (7%) (c)
Thessaloniki (41° N) Measured: 298 NIWA-UKCA, WE: 318 (+7%) NIWA-UKCA, WA: 320 (+7%) GEOS-CCM, WA: 305 (+2%) (d)
Obs. Haute-Provence (44° N) Measured: 292 NIWA-UKCA, WE: 326 (+12%) NIWA-UKCA, WA: 328 (+12%) GEOS-CCM, WA: 317 (+9%) (e)
Hoher Sonnbl. (47° N) Measured: 290 NIWA-UKCA, WE: 333 (+15%) NIWA-UKCA, WA: 336 (+16%) GEOS-CCM, WA: 328 (+13%) (f)
Villeneuve-dAscq (51° N) Measured: 293 NIWA-UKCA, WE: 342 (+17%) NIWA-UKCA, WA: 344 (+17%) GEOS-CCM, WA: 341 (+16%) (g)
Barrow (71° N) Measured: 321 NIWA-UKCA, WE: 346 (+8%) NIWA-UKCA, WA: 354 (+10%) GEOS-CCM, WA: 355 (+11%) (h)
Summit (73° N) Measured: 287 NIWA-UKCA, WE: 344 (+20%) NIWA-UKCA, WA: 352 (+23%) GEOS-CCM, WA: 354 (+23%) (i)
Saint-Denis (21° S) Measured: 260 NIWA-UKCA, WE: 280 (+8%) NIWA-UKCA, WA: 281 (+8%) GEOS-CCM, WA: 258 (-1%) (j)
Alice Springs (24° S) Measured: 260 NIWA-UKCA, WE: 281 (+8%) NIWA-UKCA, WA: 282 (+8%) GEOS-CCM, WA: 262 (+1%) (k)
Melbourne (38° S) Measured: 280 NIWA-UKCA, WE: 299 (+7%) NIWA-UKCA, WA: 302 (+8%) GEOS-CCM, WA: 292 (+4%) (l)
Lauder (45° S) Measured: 280 NIWA-UKCA, WE: 311 (+11%) NIWA-UKCA, WA: 314 (+12%) GEOS-CCM, WA: 314 (+12%) (m)
Ushuaia (55° S) Measured: 288 NIWA-UKCA, WE: 330 (+15%) NIWA-UKCA, WA: 334 (+16%) GEOS-CCM, WA: 350 (+22%) (n)
Palmer (65° S) Measured: 278 NIWA-UKCA, WE: 338 (+22%) NIWA-UKCA, WA: 339 (+22%) GEOS-CCM, WA: 365 (+31%) (o)
Arrival Heights (78° S) Measured: 291 NIWA-UKCA, WE: 302 (+4%) NIWA-UKCA, WA: 299 (+3%) GEOS-CCM, WA: 340 (+17%) (p)
South Pole (90° S) Measured: 278 NIWA-UKCA, WE: 290 (+4%) NIWA-UKCA, WA: 285 (+3%) GEOS-CCM, WA: 324 (+17%) (q)
1975 1985 1995 2005 2015

Summit Station, Greenland (72.58° N, 38.46° W, 3202 m a.s.l)

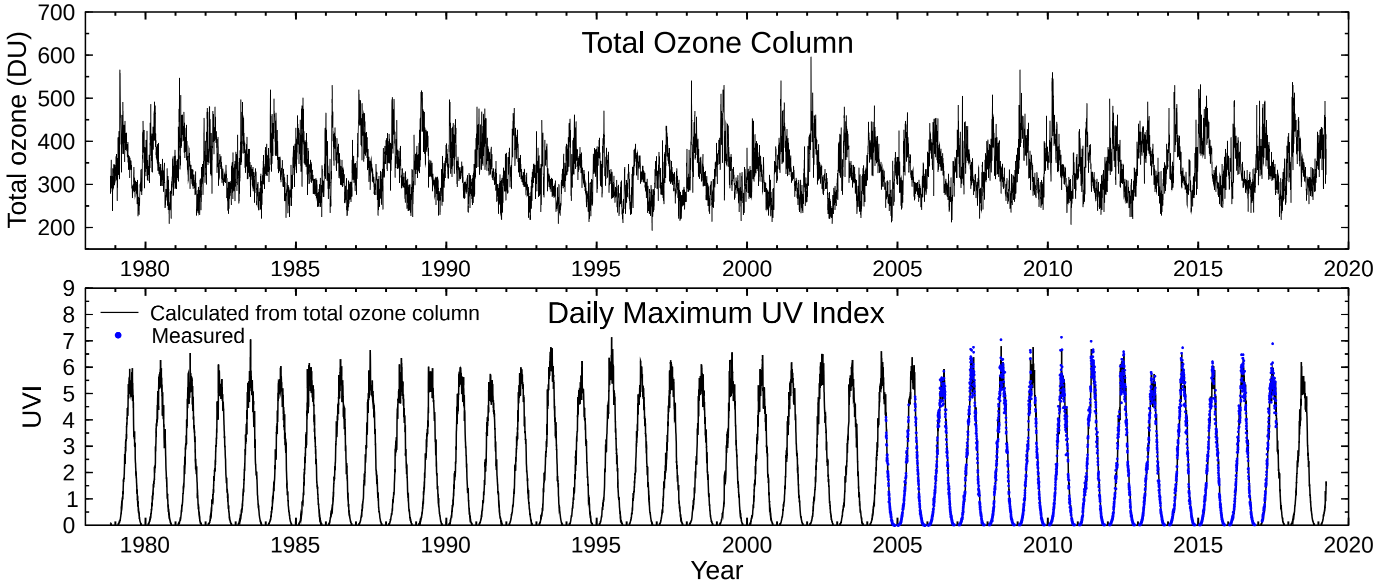

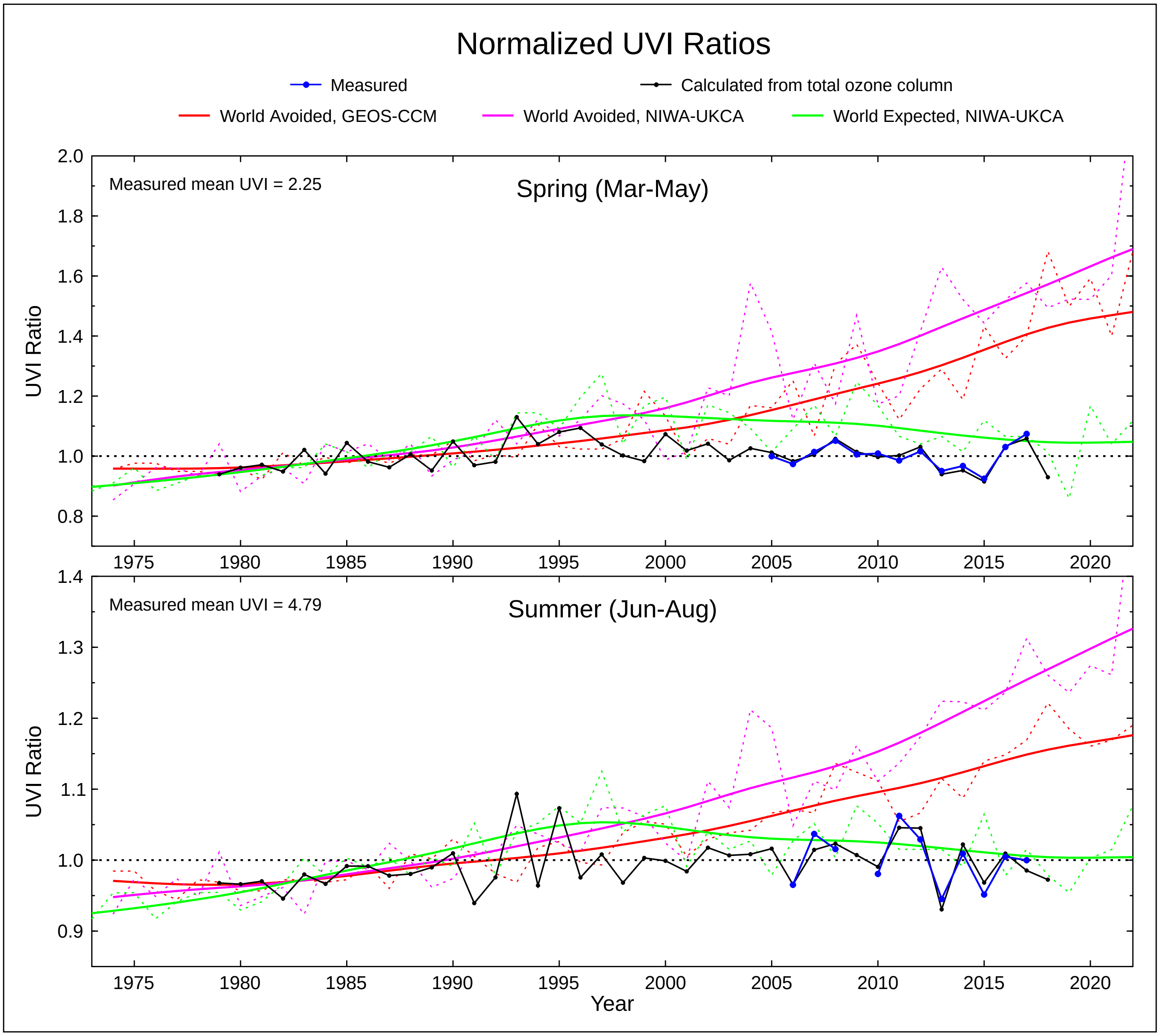

Barrow (Utqiagvik), Alaska (71.32° N, 156.68° W, 8 m a.s.l)

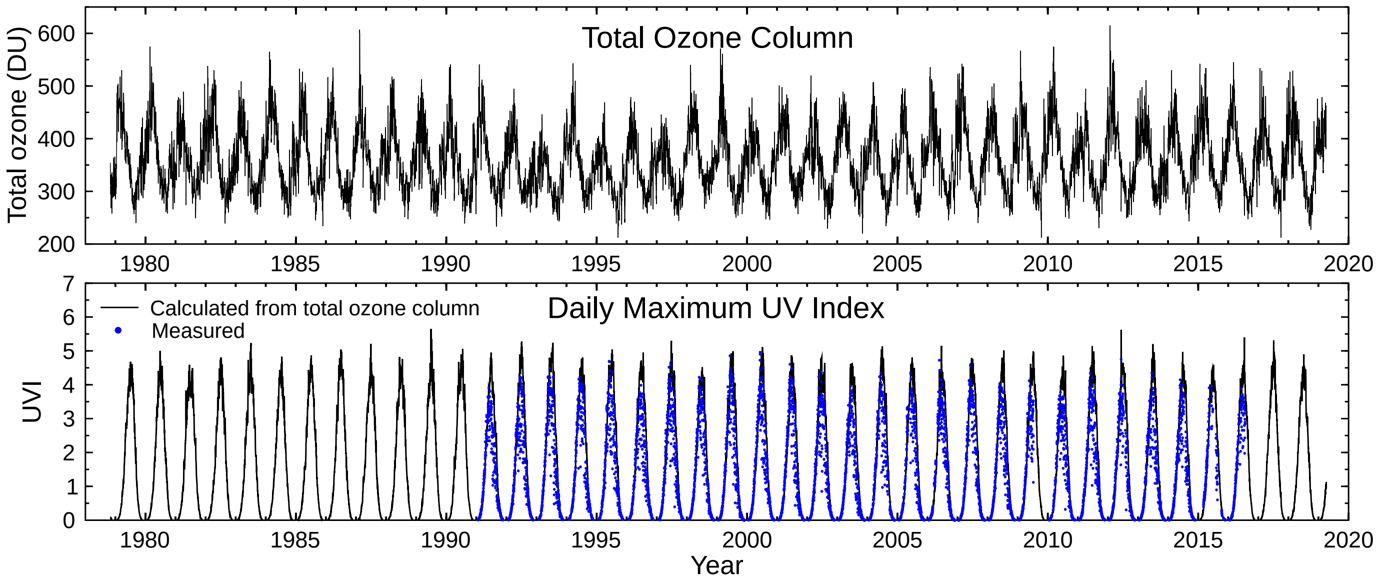

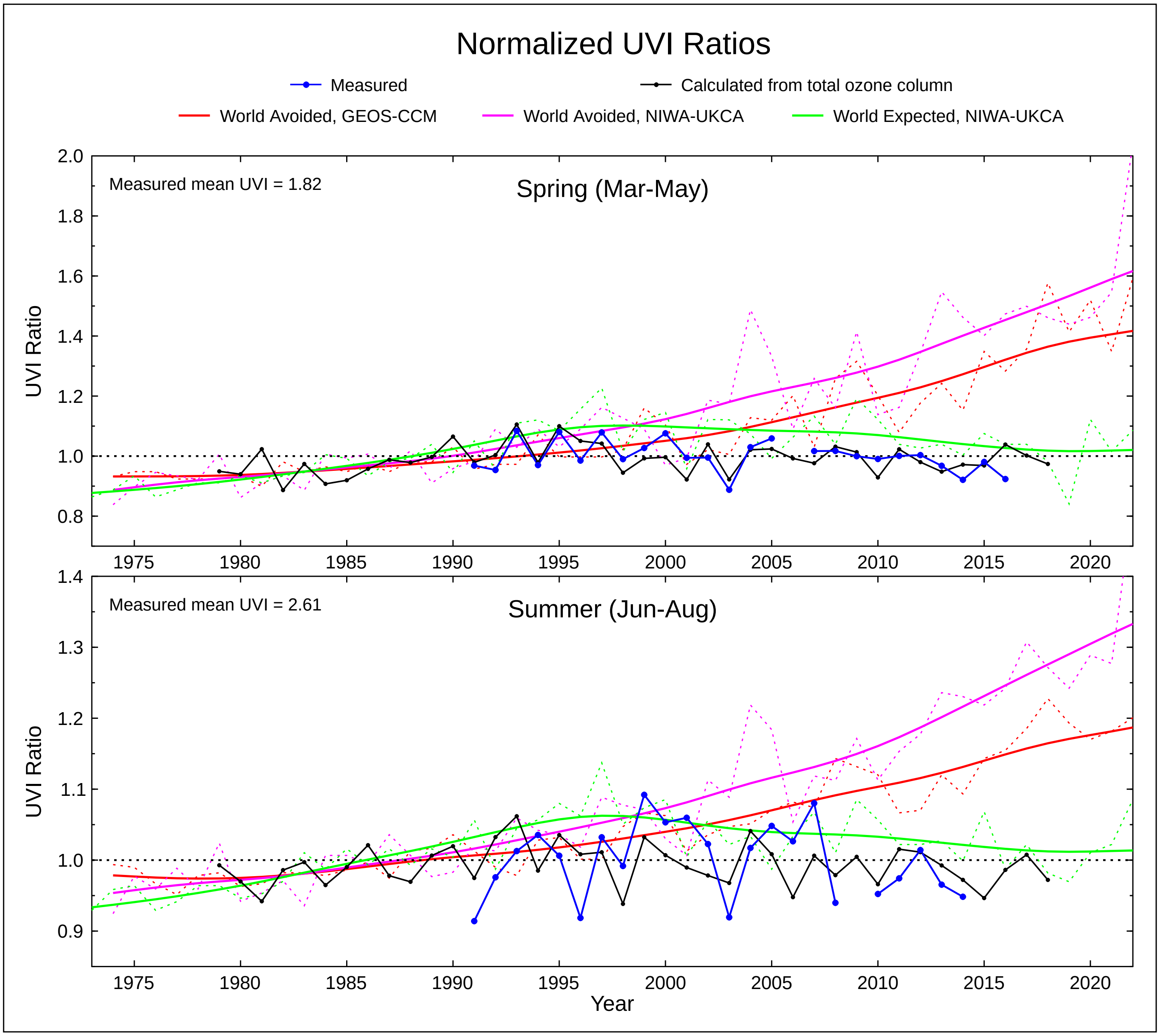

Villeneuve-d’Ascq, France (50.61° N, 3.14° E, 70 m a.s.l)

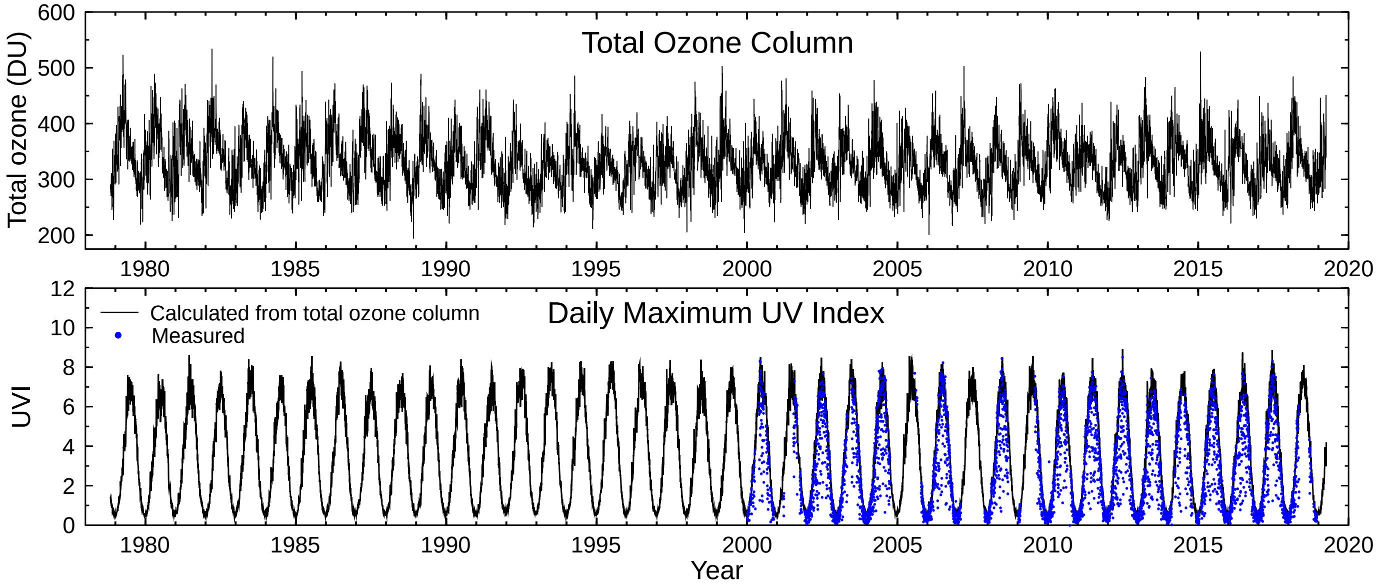

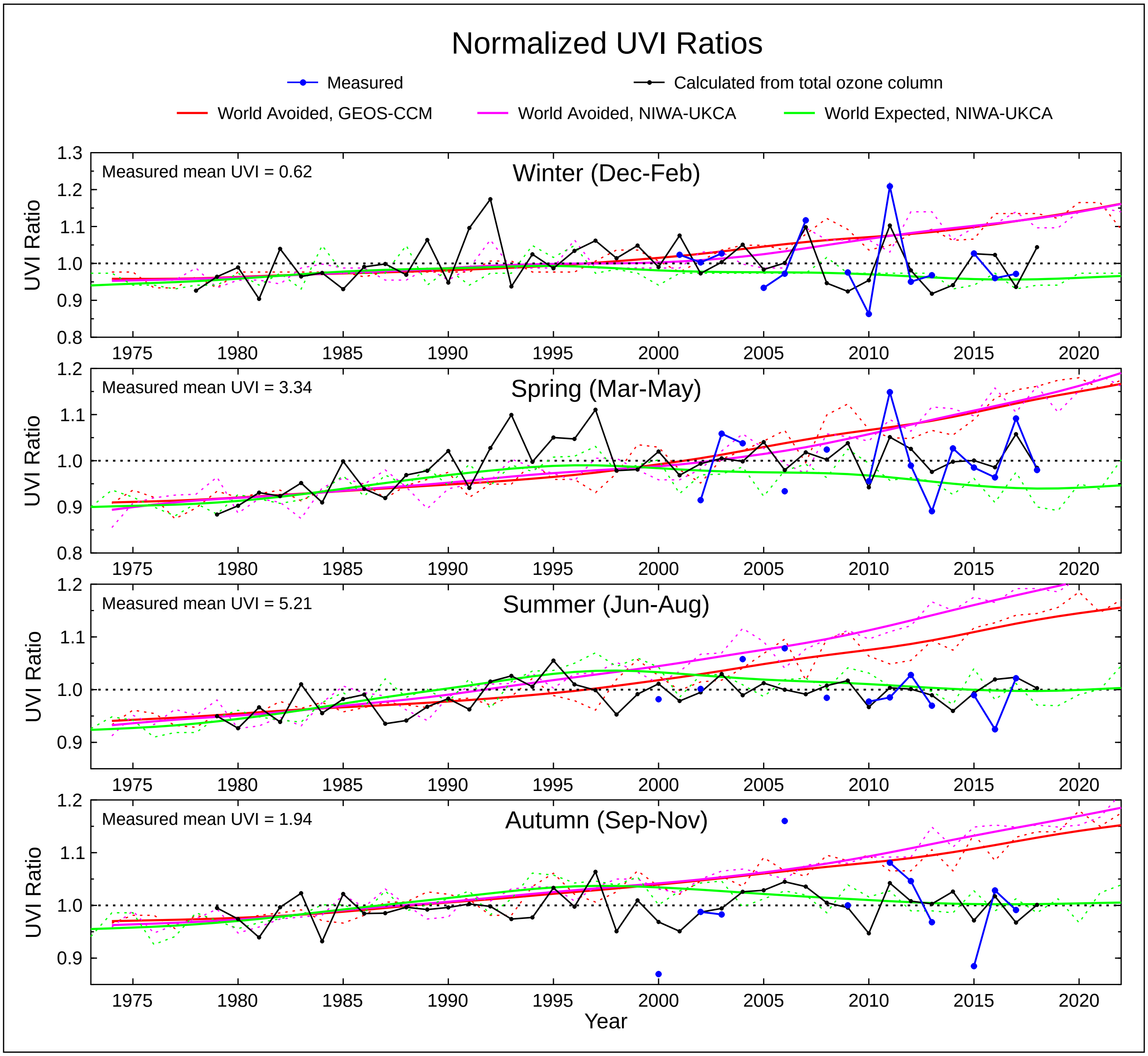

Hoher Sonnblick, Austria (47.05° N, 12.95° E, 3106 m a.s.l)

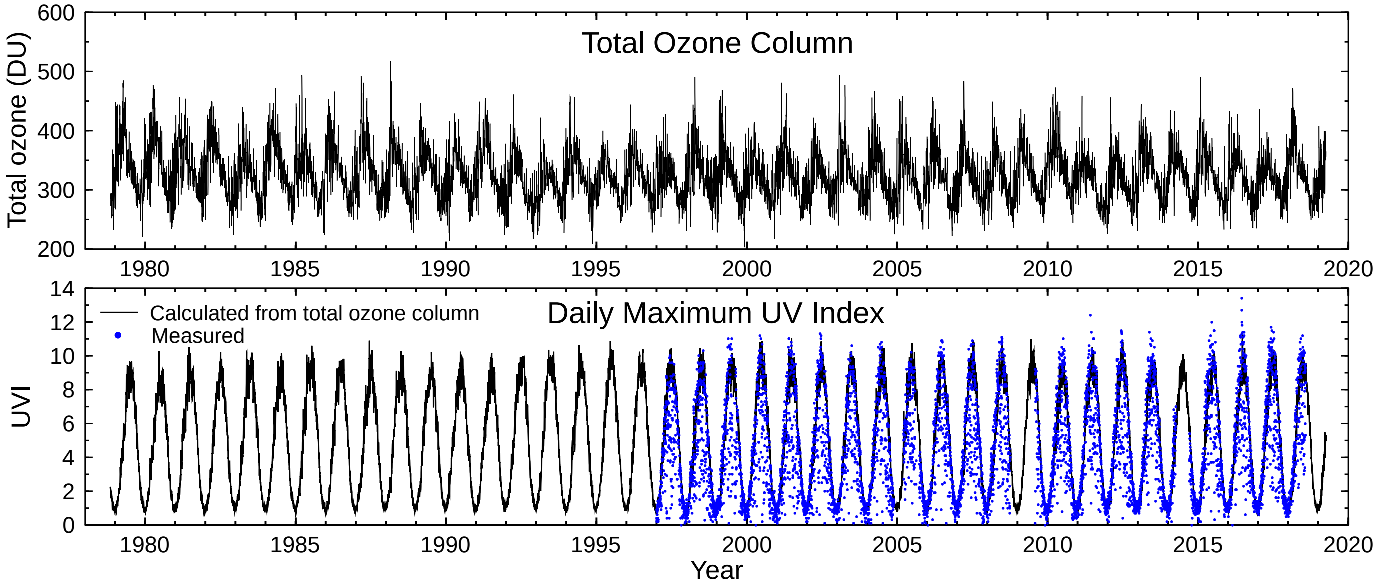

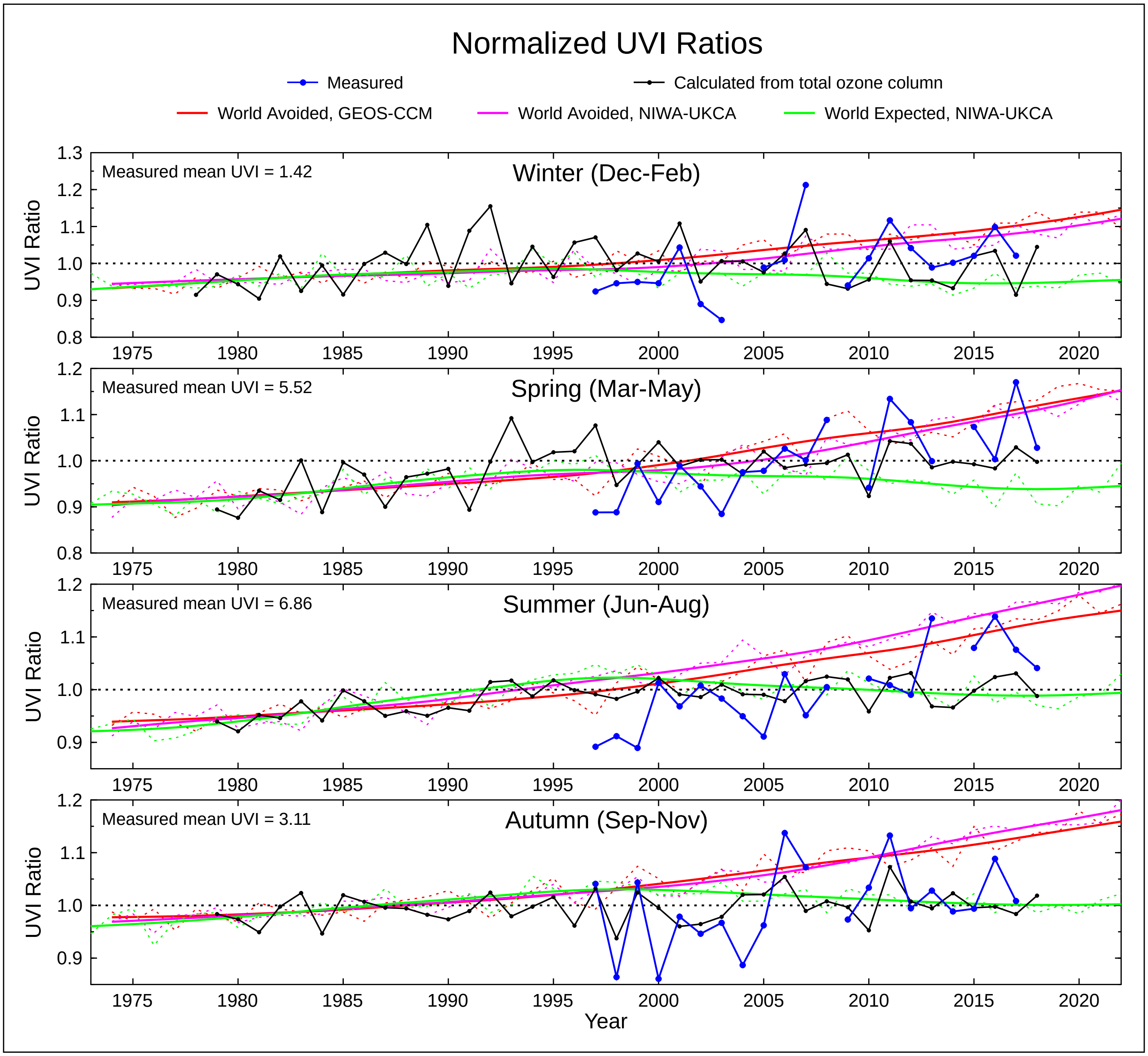

Haute-Provence Observatory, France (43.94° N, 5.72° E, 688 m a.s.l)

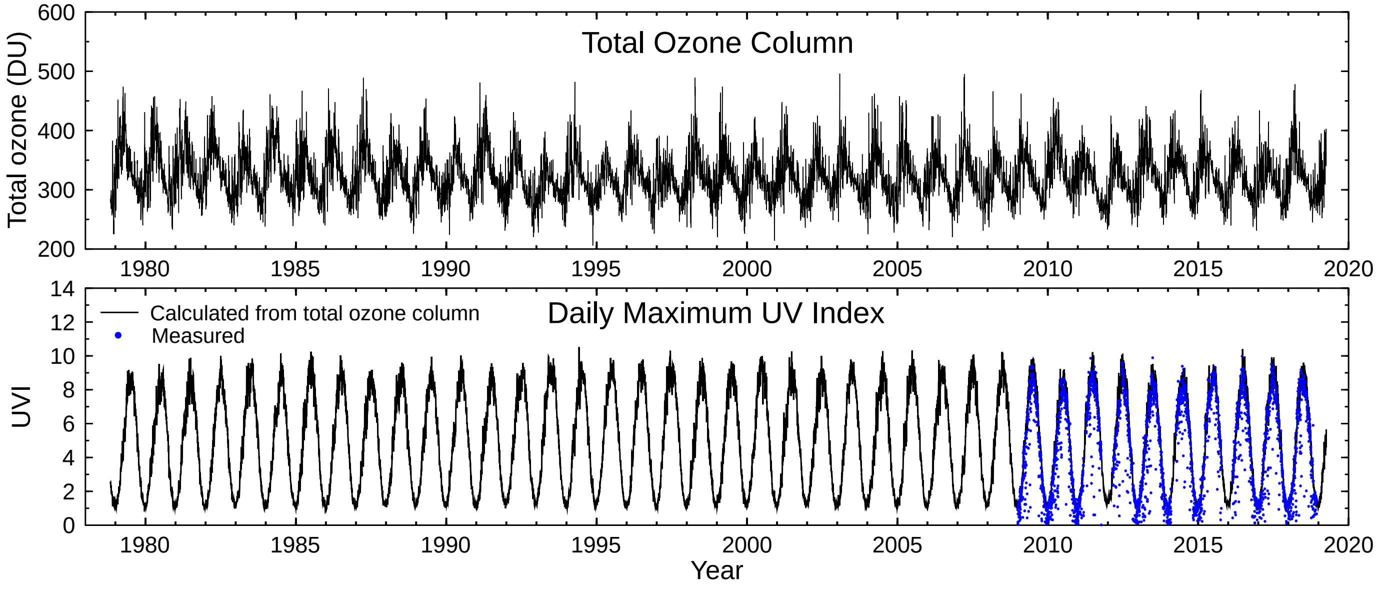

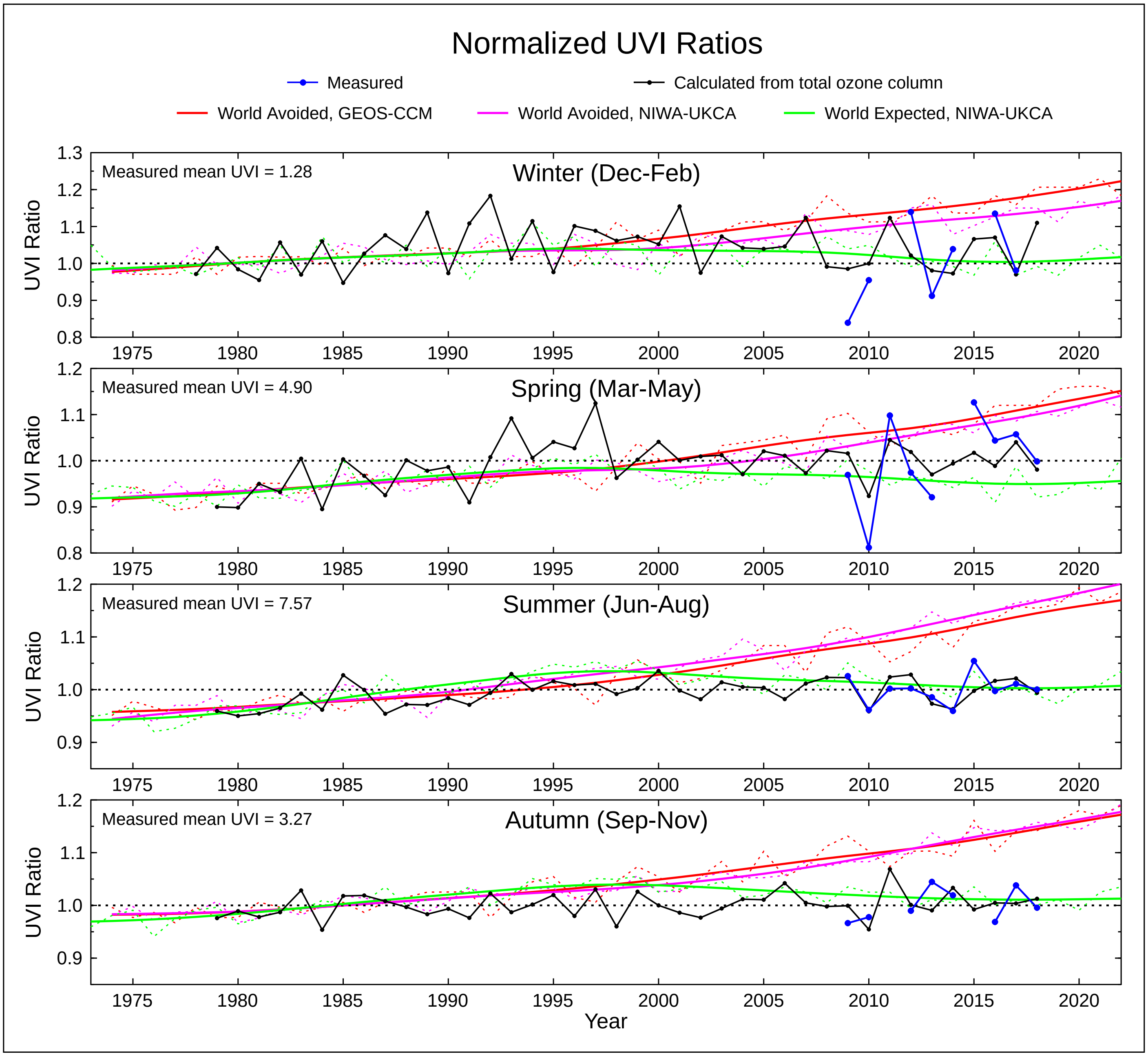

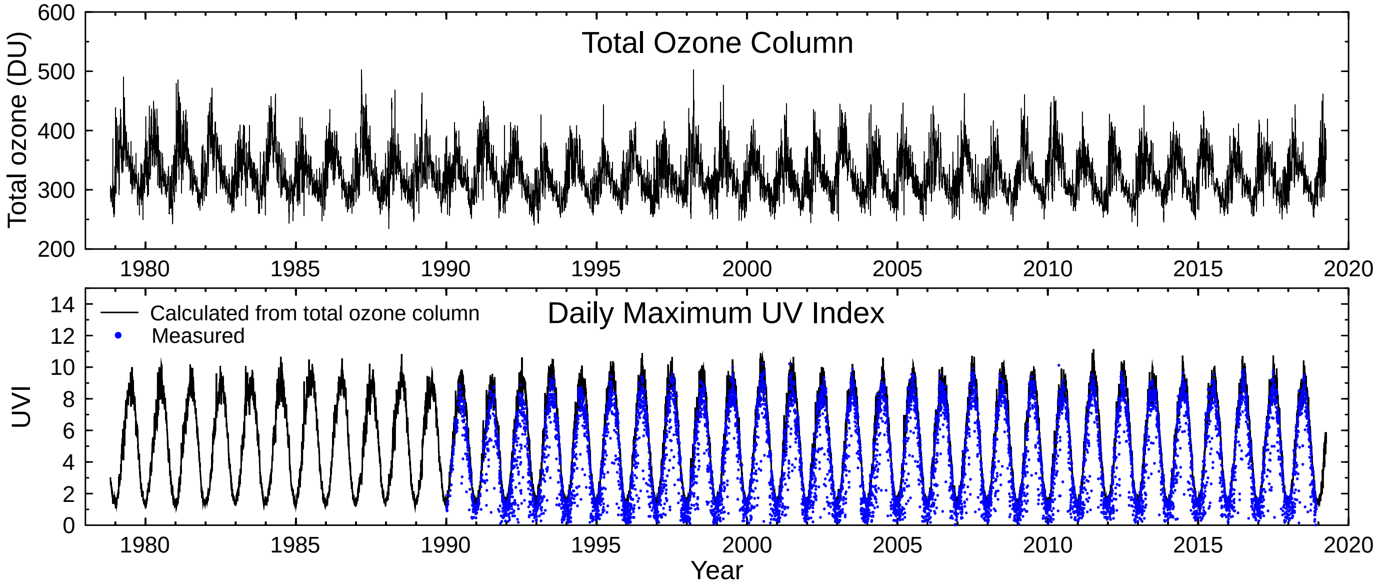
Total Ozone Column
Total ozone (DU)
Daily Maximum UV Index
Calculated from total ozone column
Measured
UVI
Year

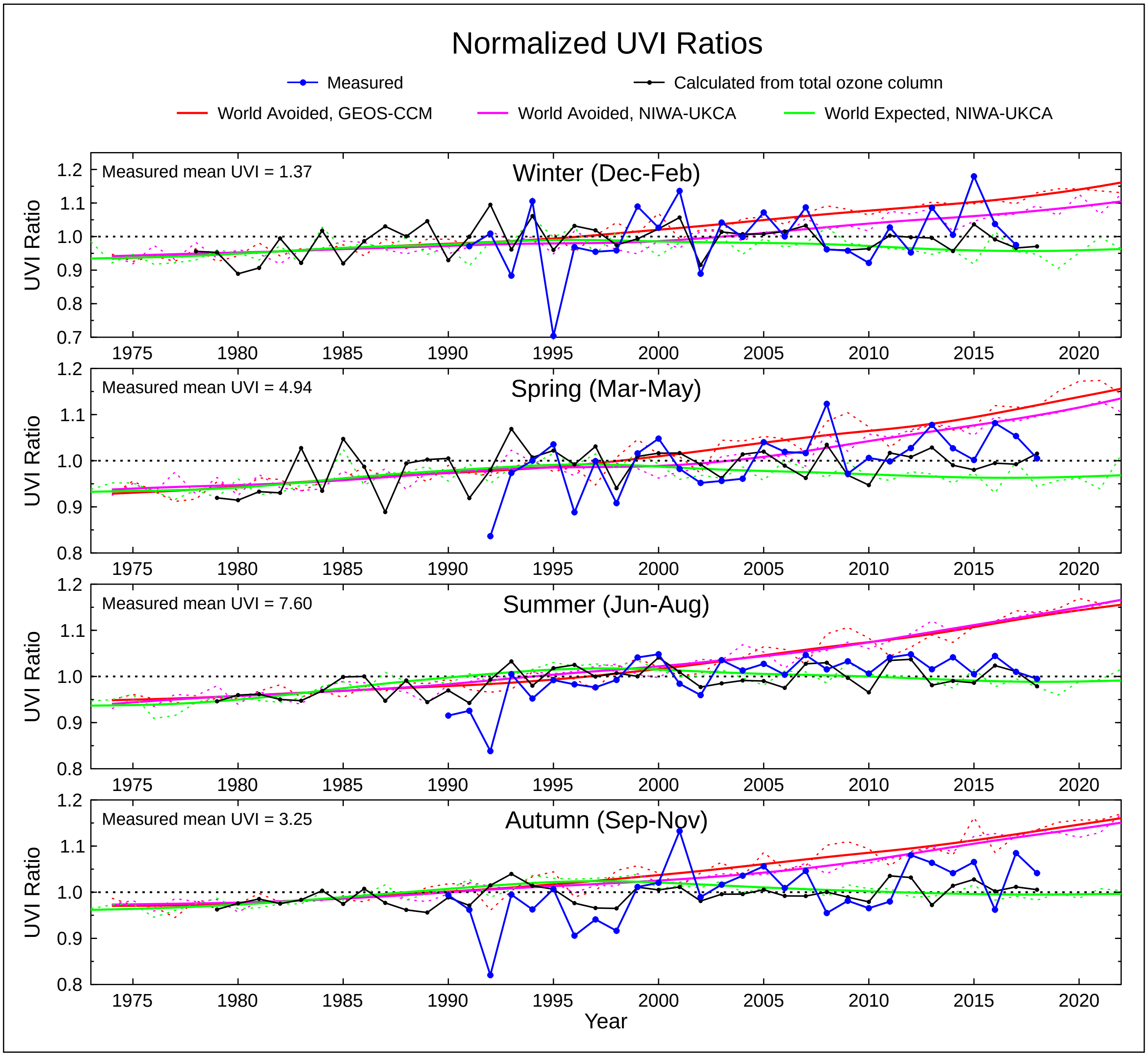
Normalized UVI Ratios
Measured
Calculated from total ozone column
World Avoided, GEOS-CCM
World Avoided, NIWA-UKCA
World Expected, NIWA-UKCA
Measured mean UVI = 1.37
Winter (Dec-Feb)
Measured mean UVI = 4.94
Spring (Mar-May)
Measured mean UVI = 7.60
Summer (Jun-Aug)
Measured mean UVI = 3.25
Autumn (Sep-Nov)
UVI Ratio
Year

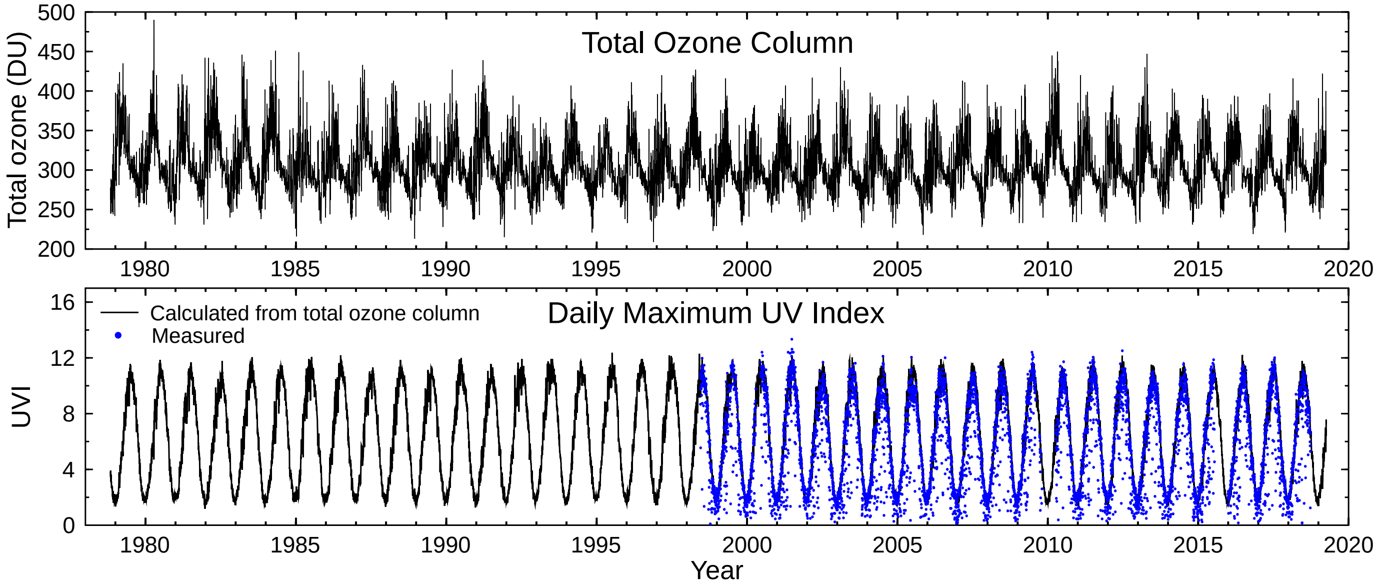
Total Ozone Column
Total ozone (DU)
500
450
400
350
300
250
200
1980
1985
1990
1995
2000
2005
2010
2015
2020
Daily Maximum UV Index
Calculated from total ozone column
Measured
UVI
16
12
8
4
0
Year

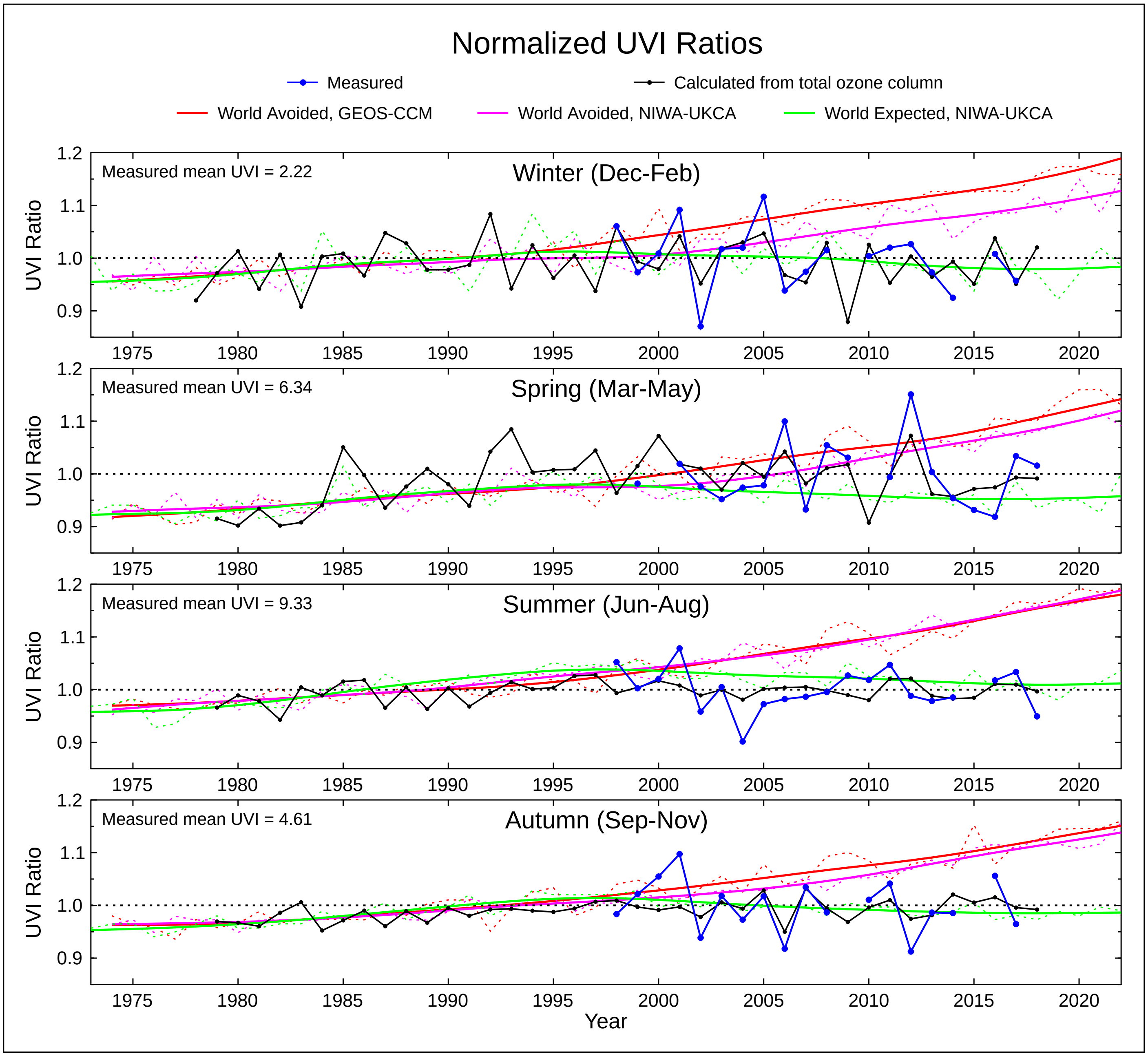
Normalized UVI Ratios
Measured
Calculated from total ozone column
World Avoided, GEOS-CCM
World Avoided, NIWA-UKCA
World Expected, NIWA-UKCA
Measured mean UVI = 2.22
Winter (Dec-Feb)
Measured mean UVI = 6.34
Spring (Mar-May)
Measured mean UVI = 9.33
Summer (Jun-Aug)
Measured mean UVI = 4.61
Autumn (Sep-Nov)
UVI Ratio
1.2
1.1
1.0
0.9
1975
1980
1985
1990
1995
2000
2005
2010
2015
2020
Year

San Diego, USA (32.77° N, 117.20° W, 22 m a.s.l)

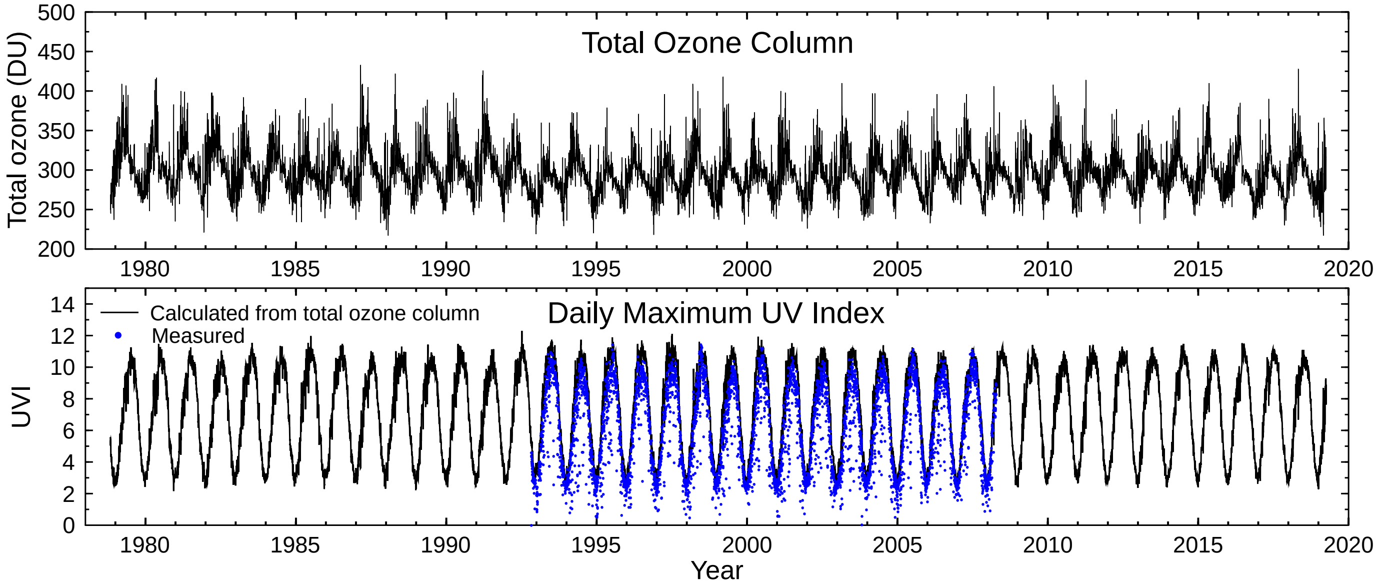

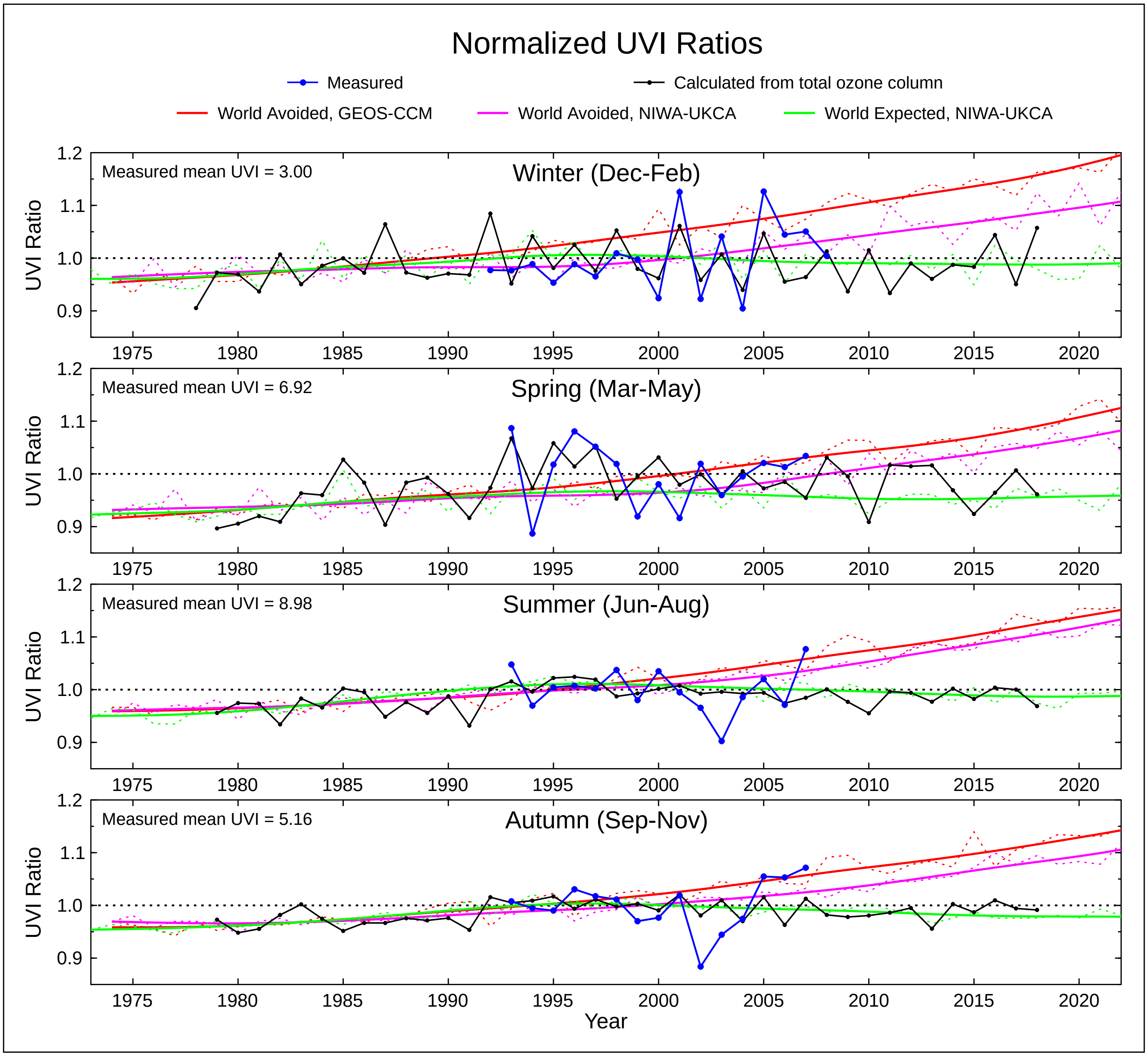

Mauna Loa Observatory, Hawaii (19.53° N, 155.57° W, 3400 m a.s.l)

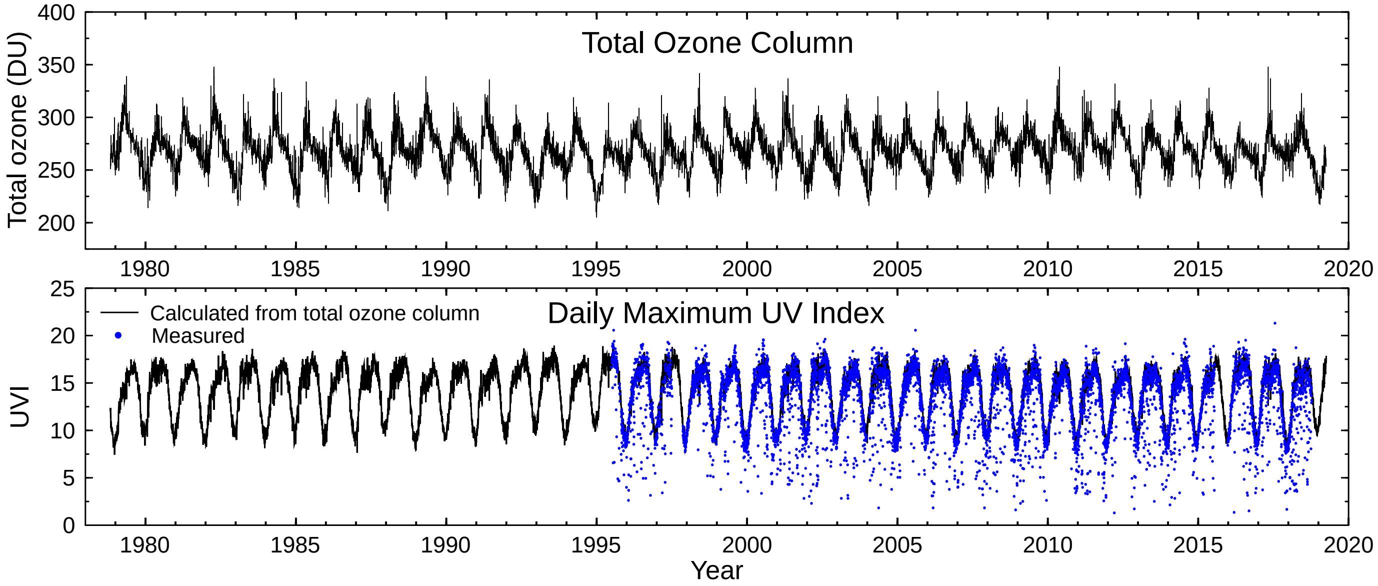

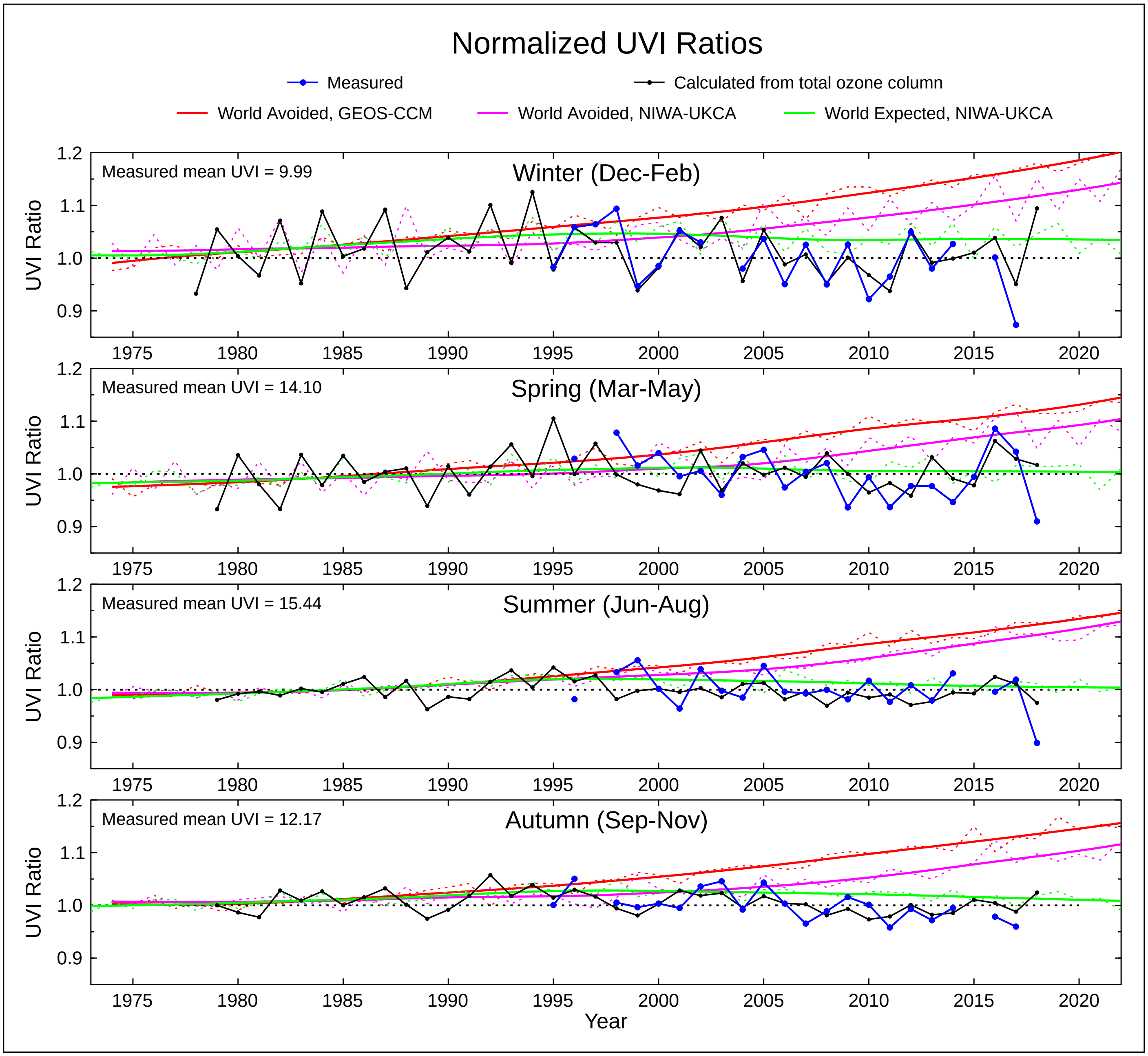

Saint-Denis, Reunion Island (20.90° S, 55.48° E, 80 m a.s.l)

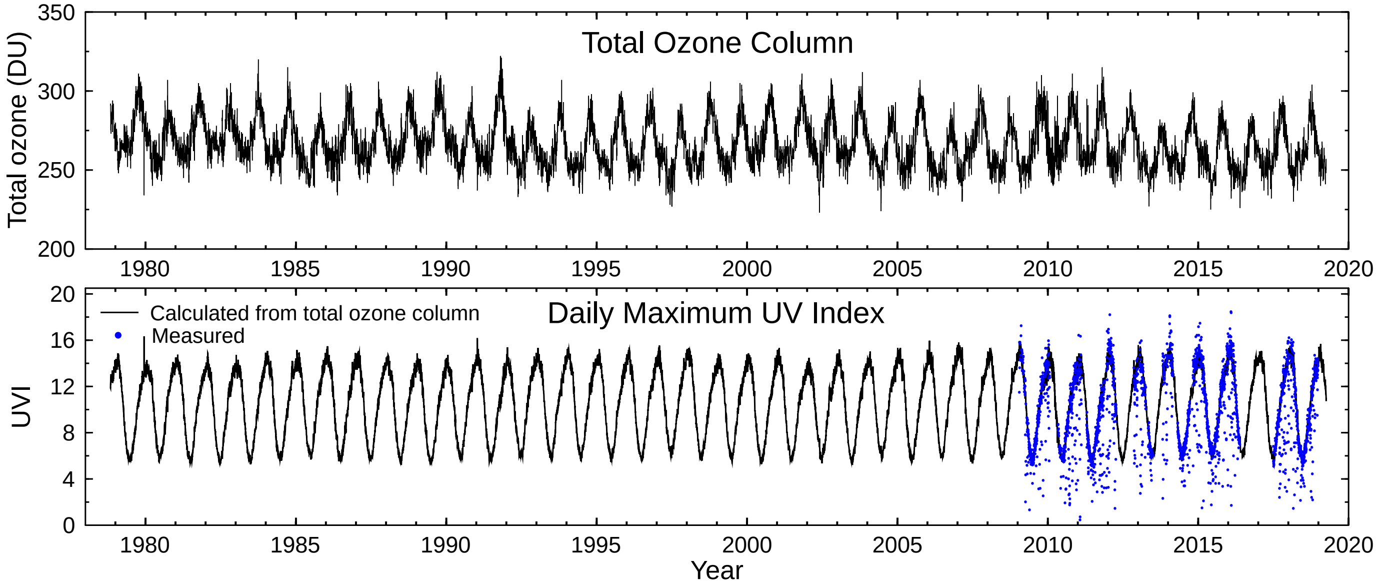

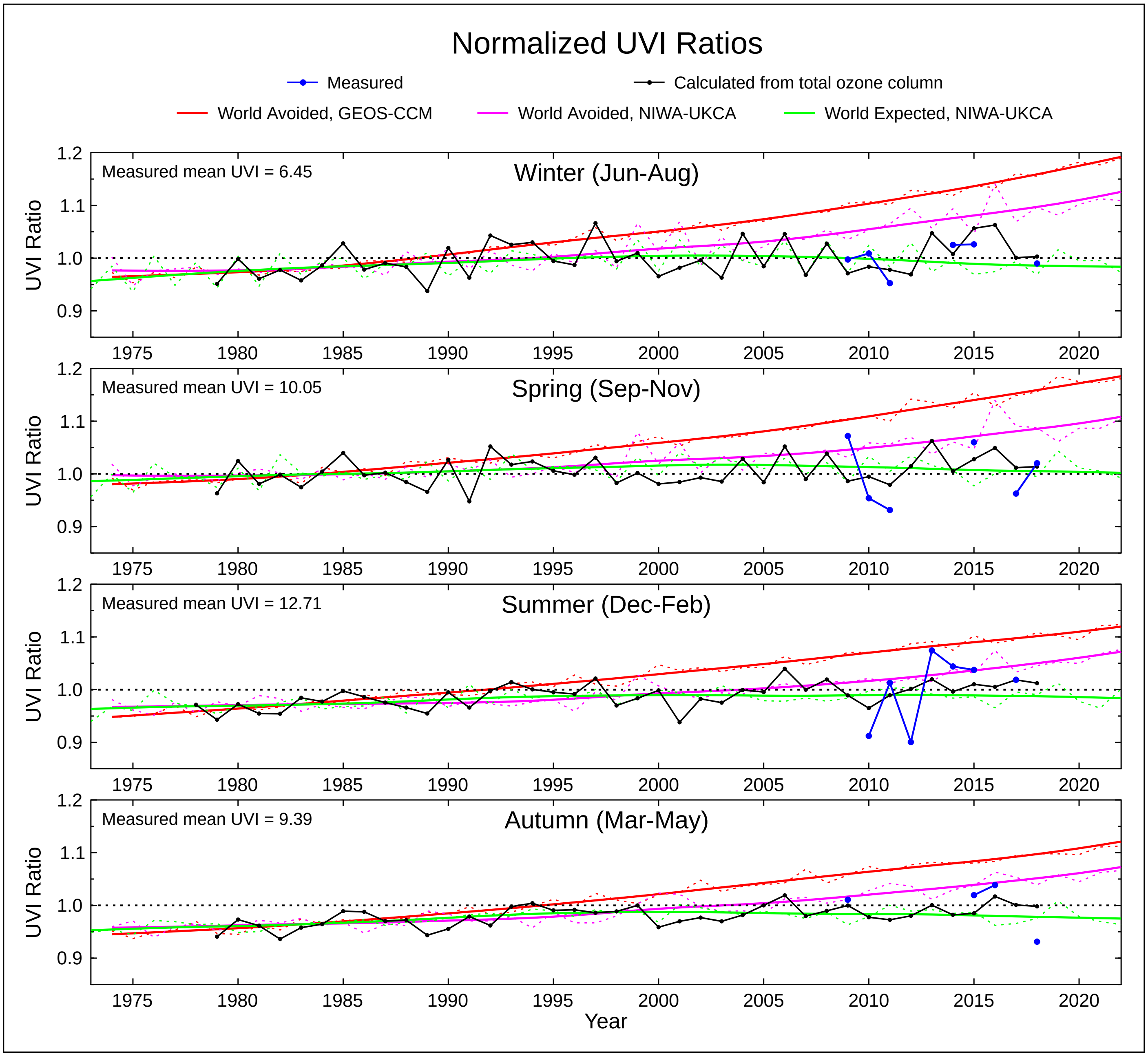

Alice Springs, Australia (23.80° S, 133.89° E, 547 m a.s.l)

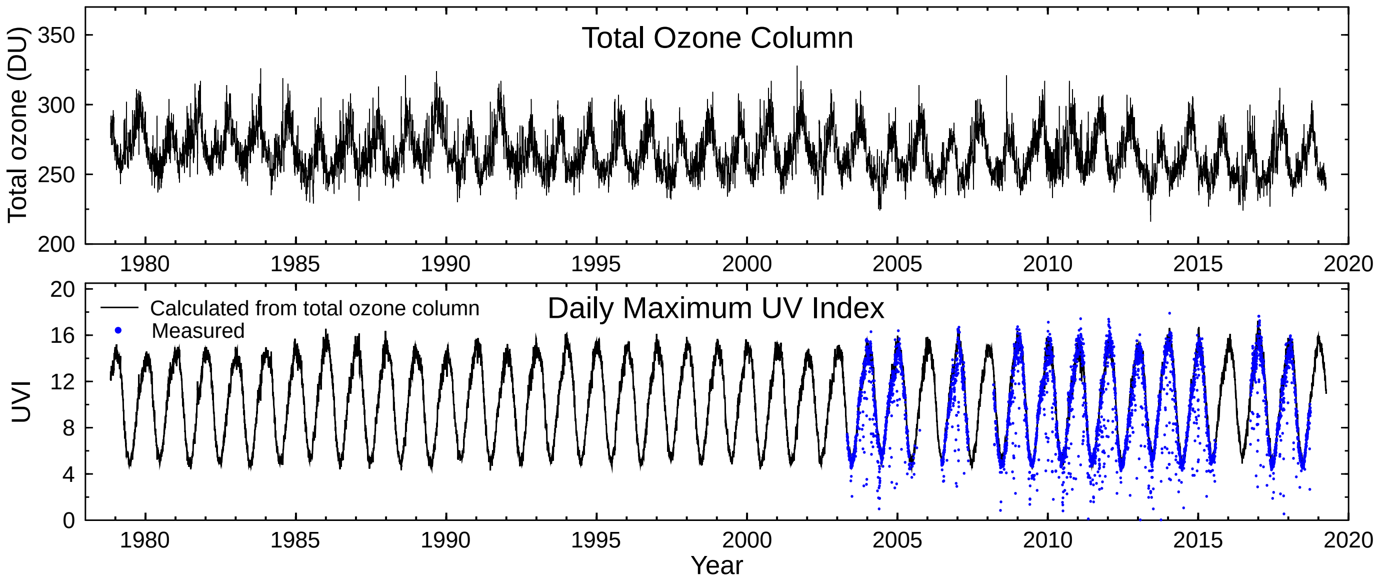

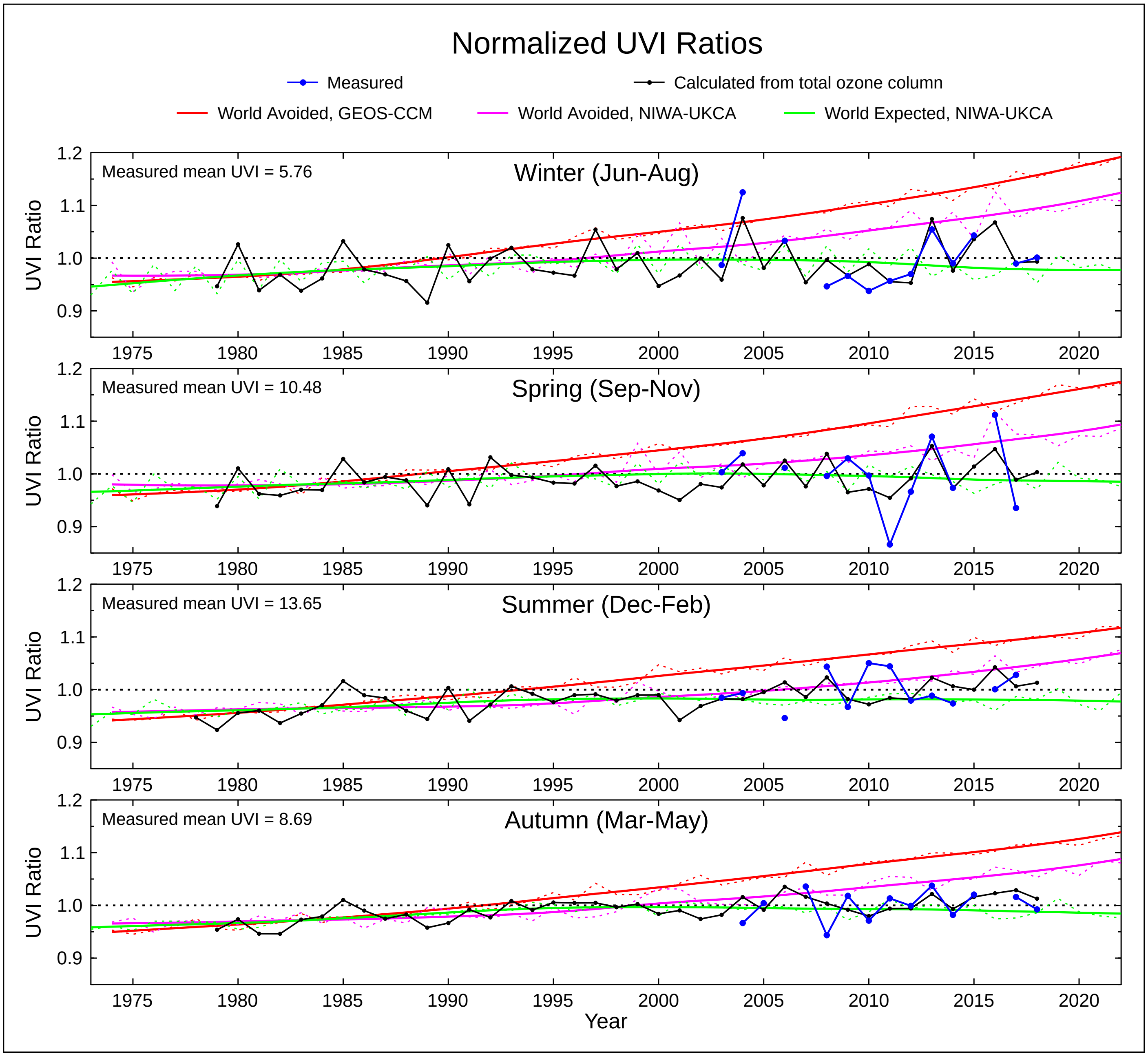

Melbourne, Australia (37.69° S, 144.94° E, 110 m a.s.l)

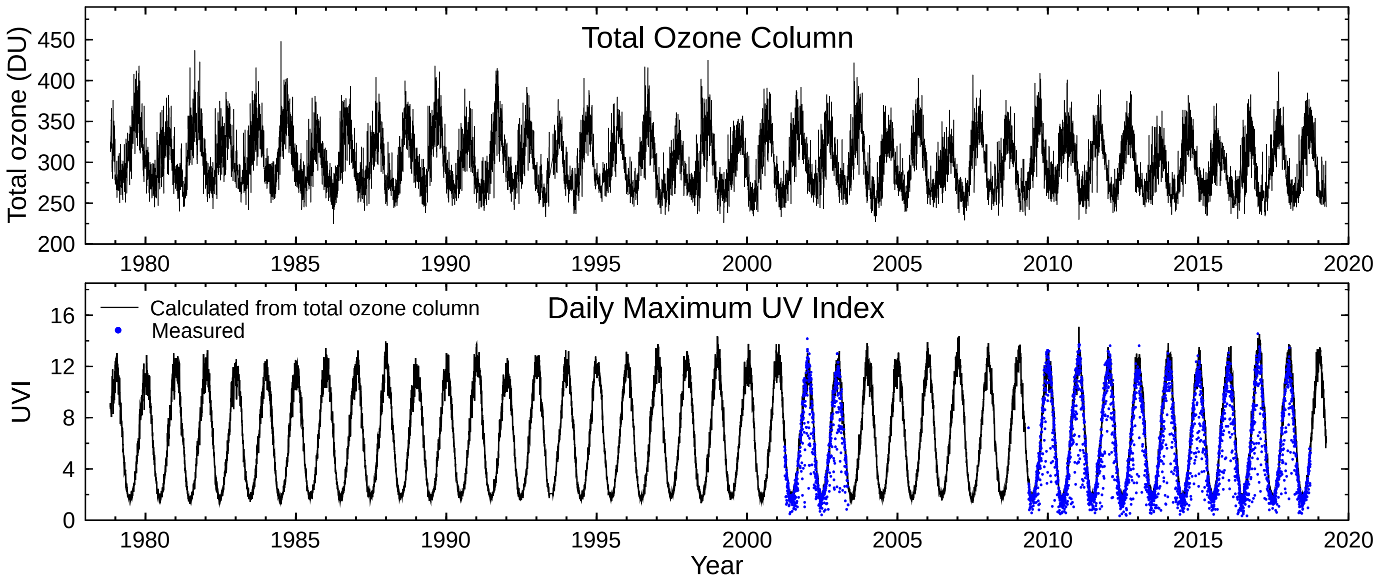

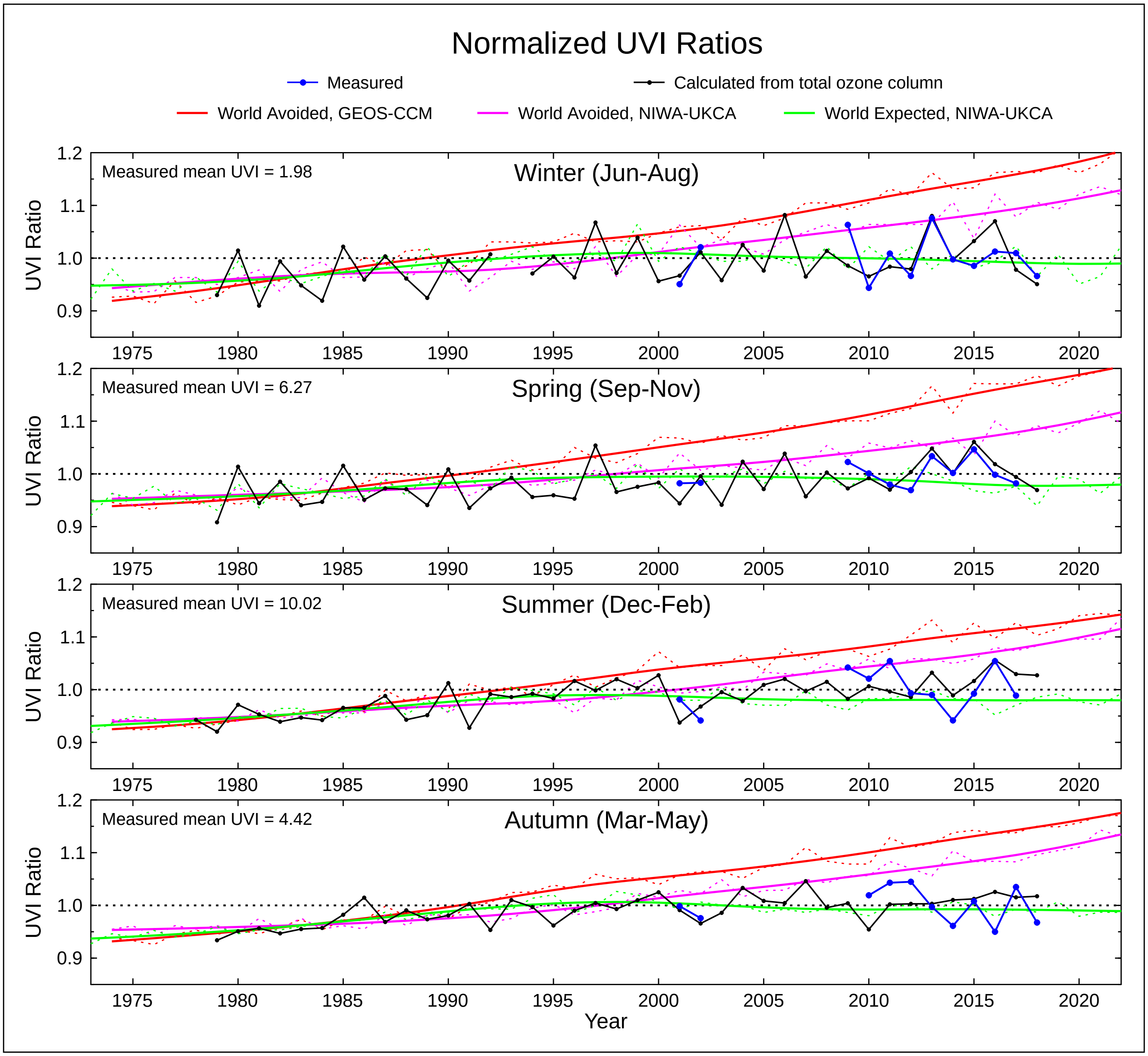

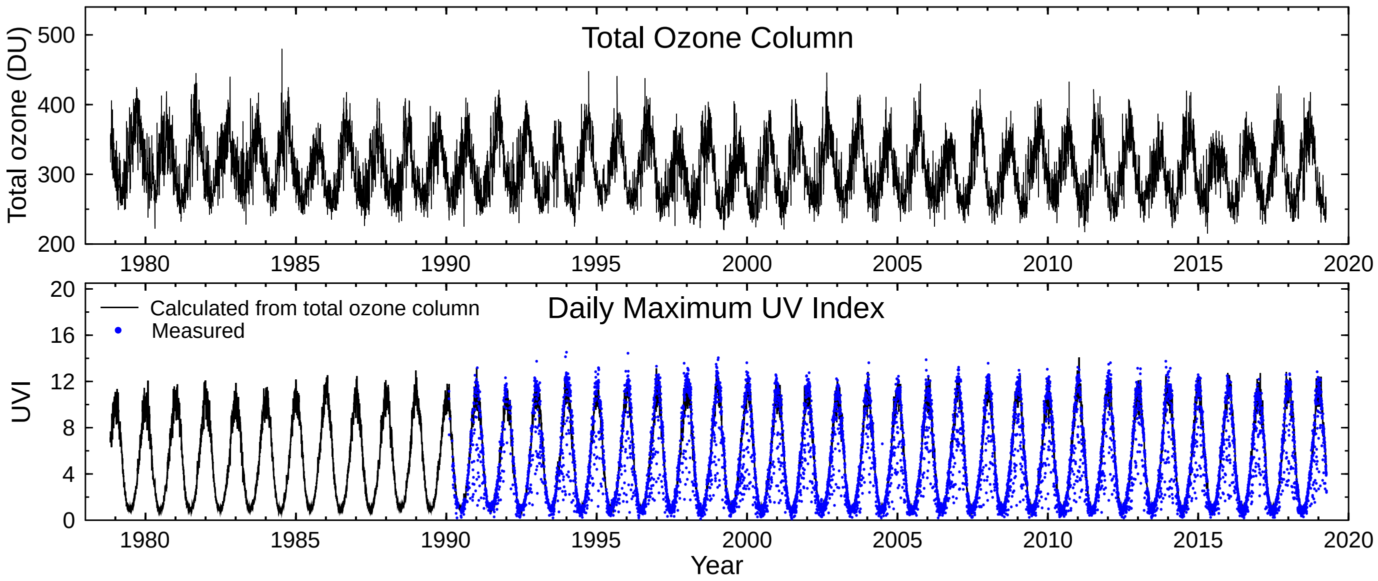
Total Ozone Column
Total ozone (DU)
Daily Maximum UV Index
Calculated from total ozone column
Measured
UVI
Year

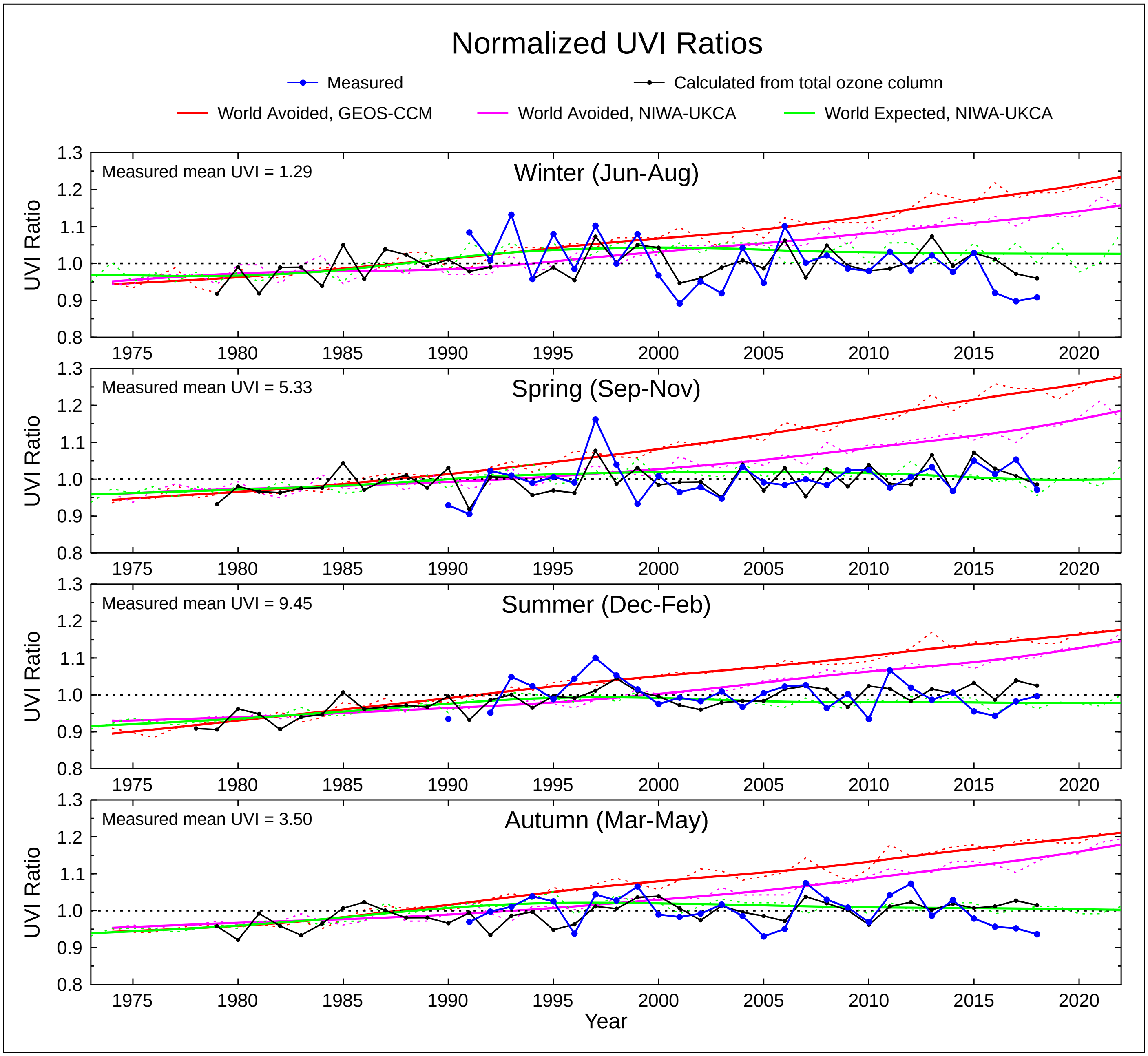
Normalized UVI Ratios
Measured
Calculated from total ozone column
World Avoided, GEOS-CCM
World Avoided, NIWA-UKCA
World Expected, NIWA-UKCA
Winter (Jun-Aug)
Measured mean UVI = 1.29
Spring (Sep-Nov)
Measured mean UVI = 5.33
Summer (Dec-Feb)
Measured mean UVI = 9.45
Autumn (Mar-May)
Measured mean UVI = 3.50
UVI Ratio
Year

Ushuaia, Argentina (54.82° S, 68.32° W, 30 m a.s.l)

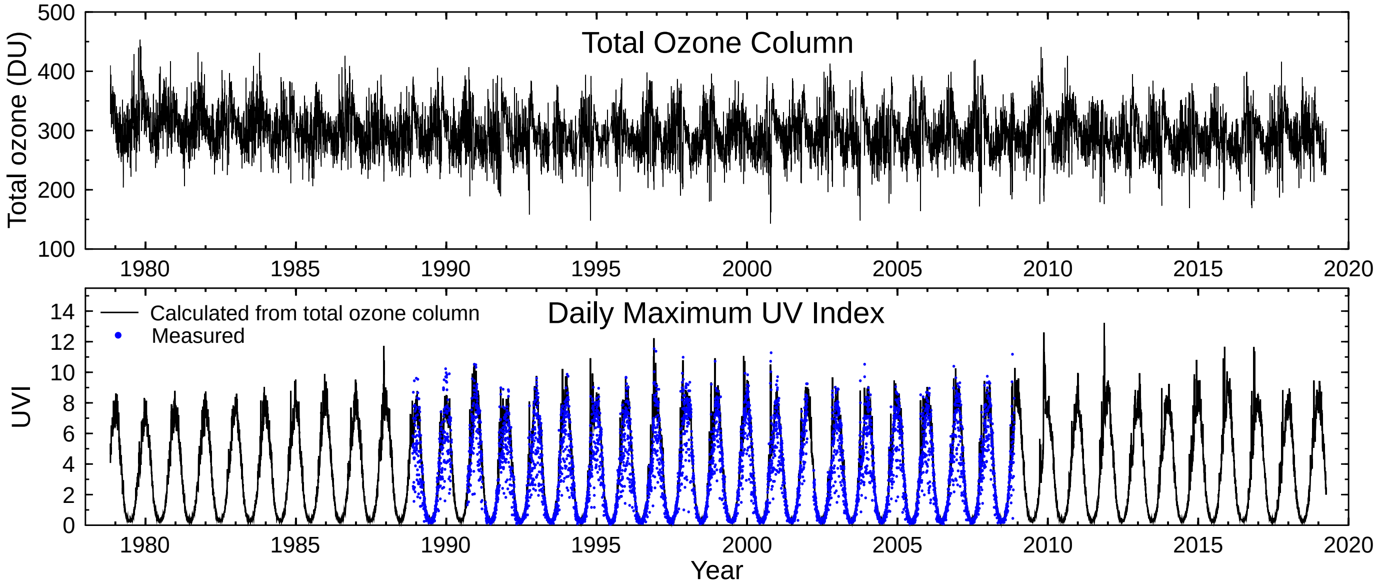

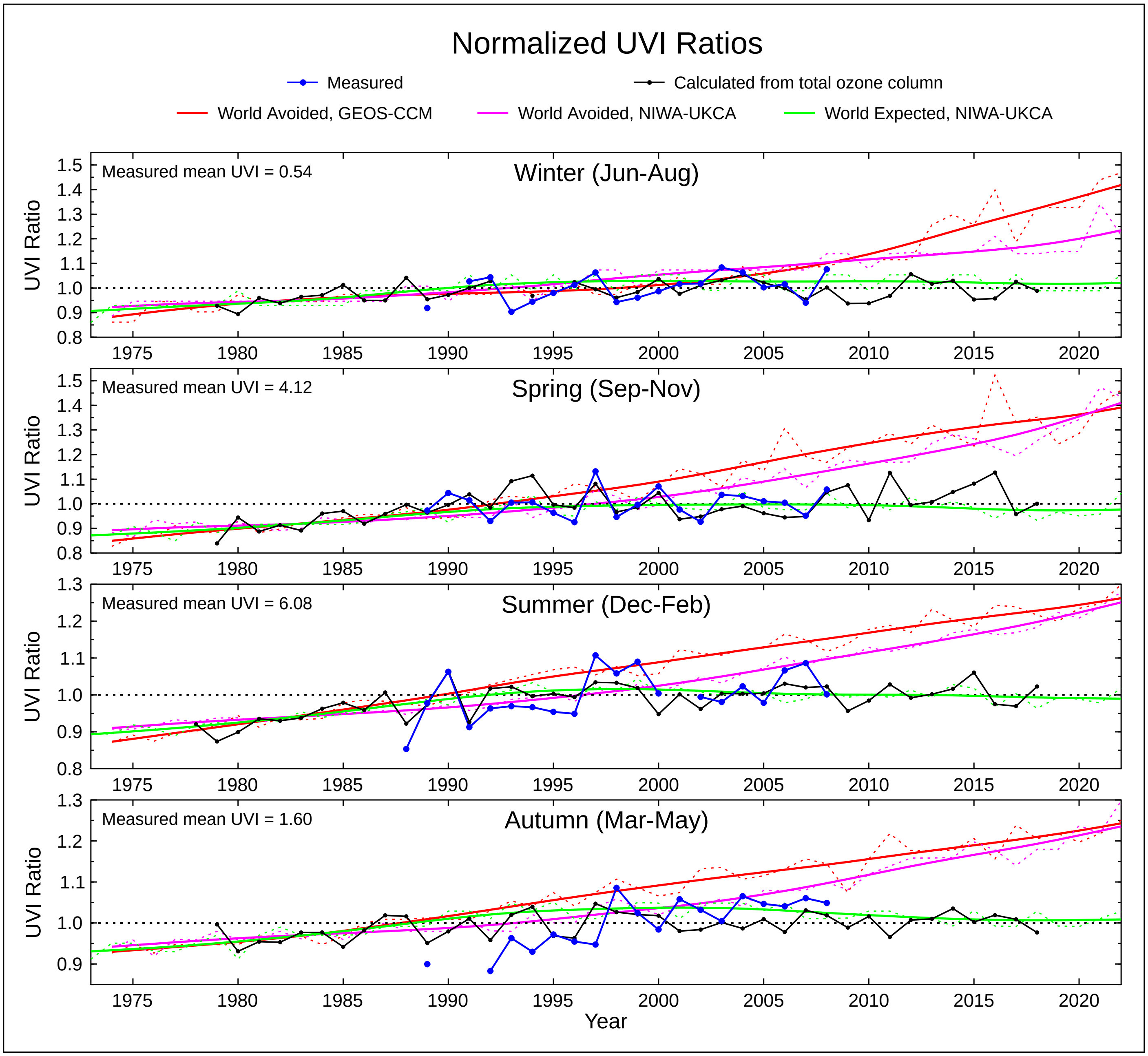

Palmer Station, Antarctica (64.77° S, 64.05° W, 21 m a.s.l)

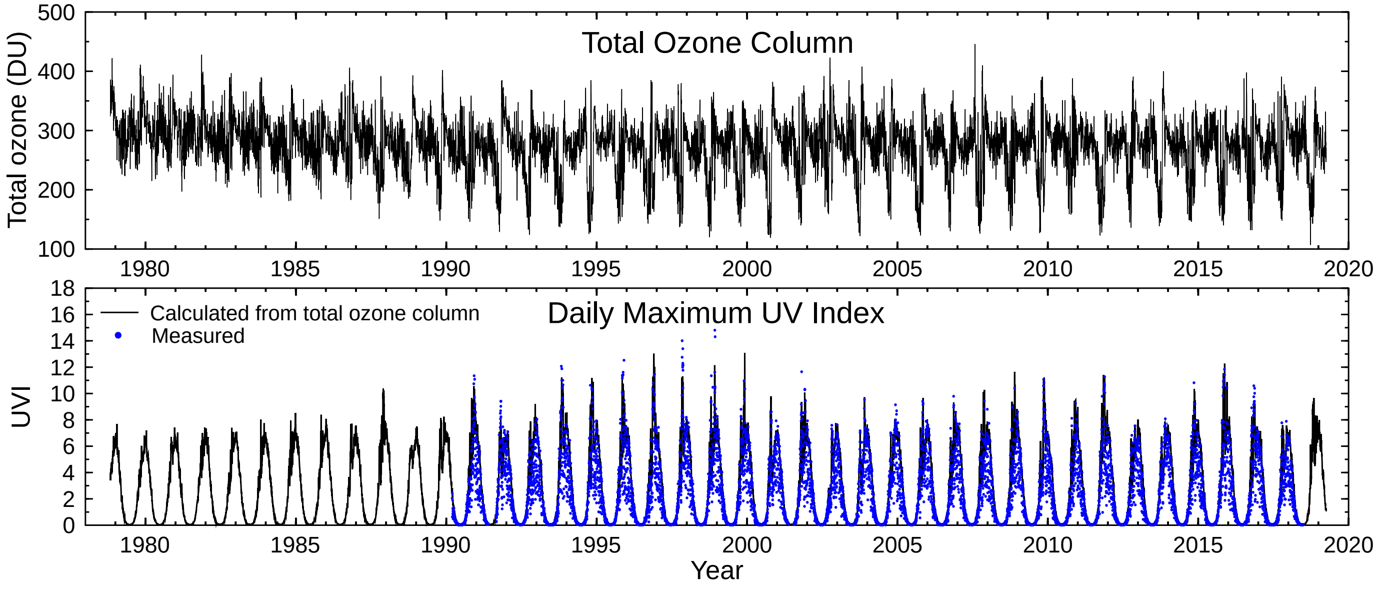

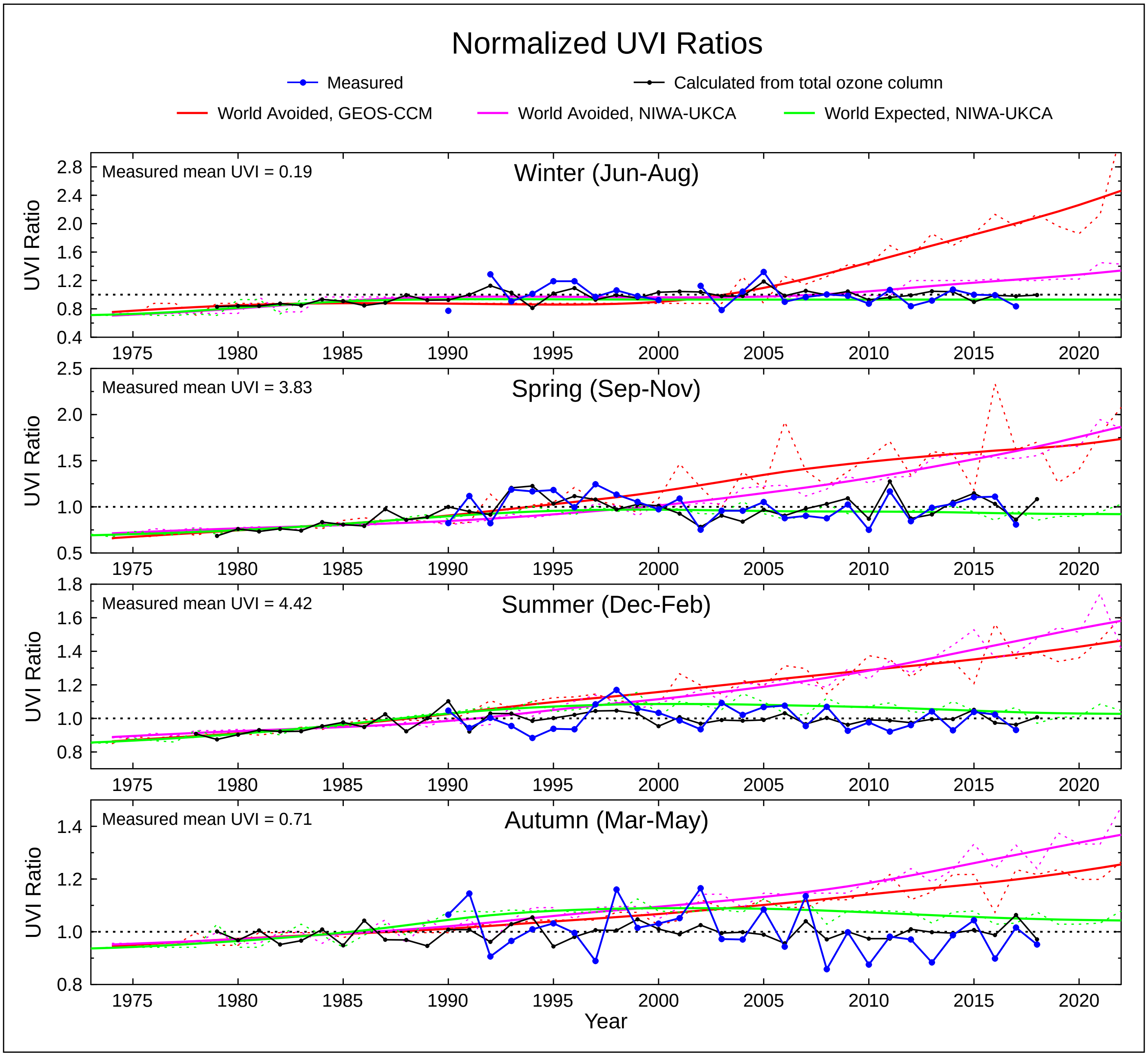

Arrival Heights, Antarctica (77.83° S, 166.66° E, 190 m a.s.l)

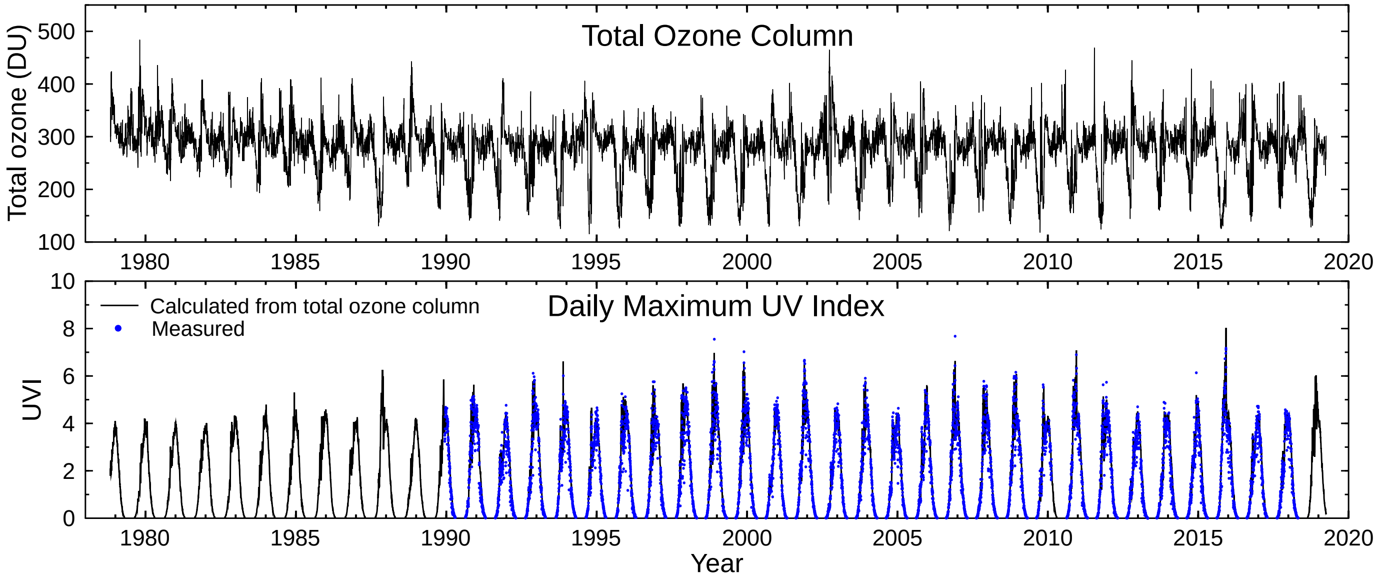

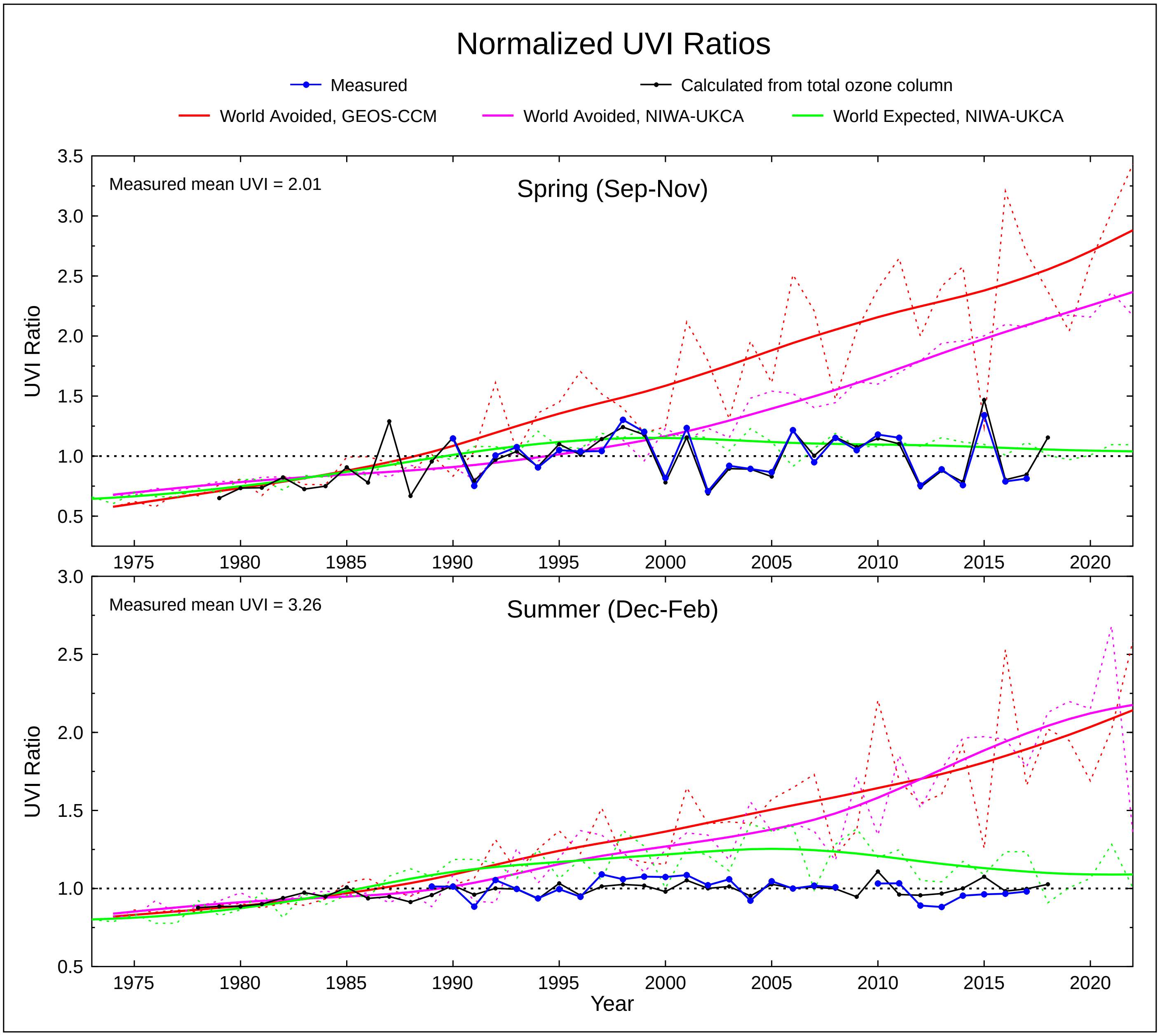

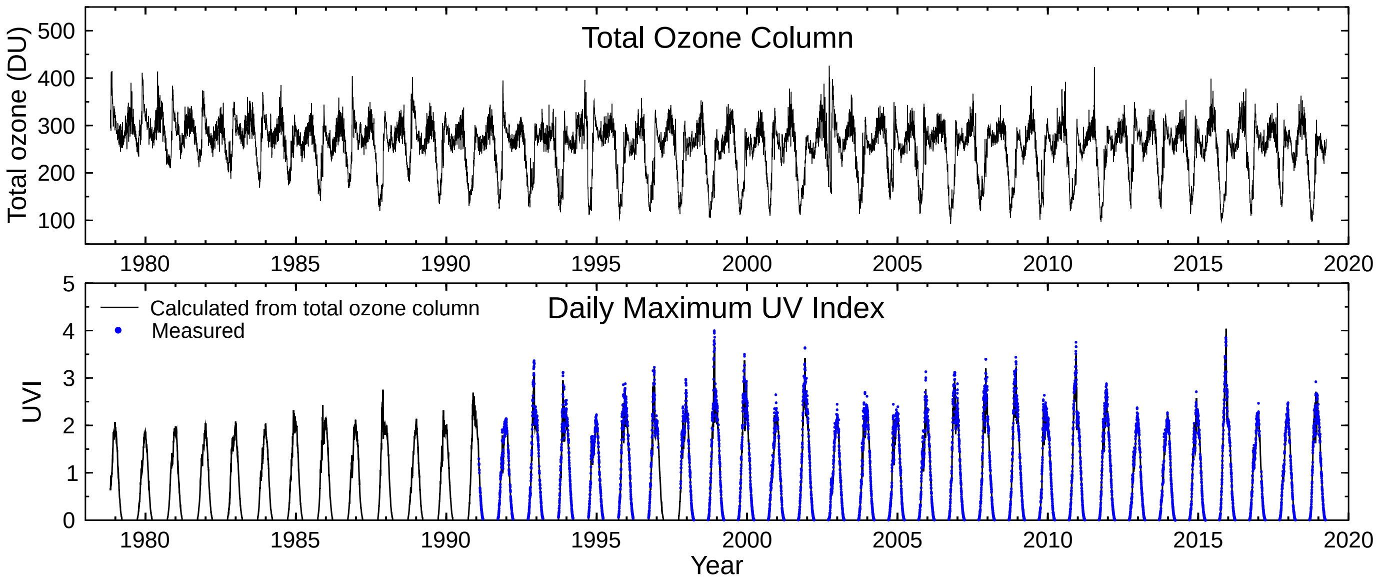
Total Ozone Column
Total ozone (DU)
Daily Maximum UV Index
Calculated from total ozone column
Measured
UVI
Year

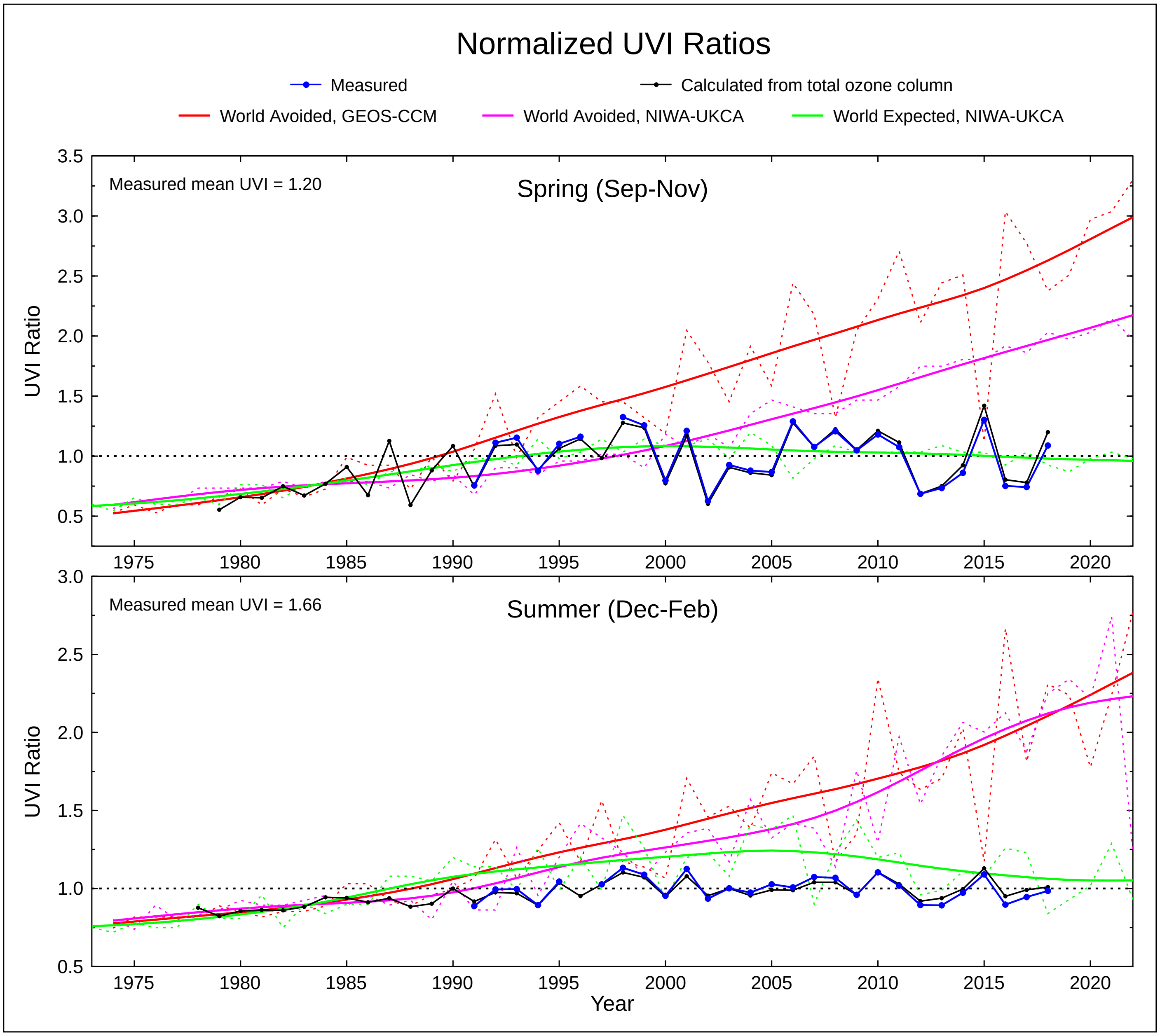
Normalized UVI Ratios
Measured
Calculated from total ozone column
World Avoided, GEOS-CCM
World Avoided, NIWA-UKCA
World Expected, NIWA-UKCA
Measured mean UVI = 1.20
Spring (Sep-Nov)
UVI Ratio
Measured mean UVI = 1.66
Summer (Dec-Feb)
Year

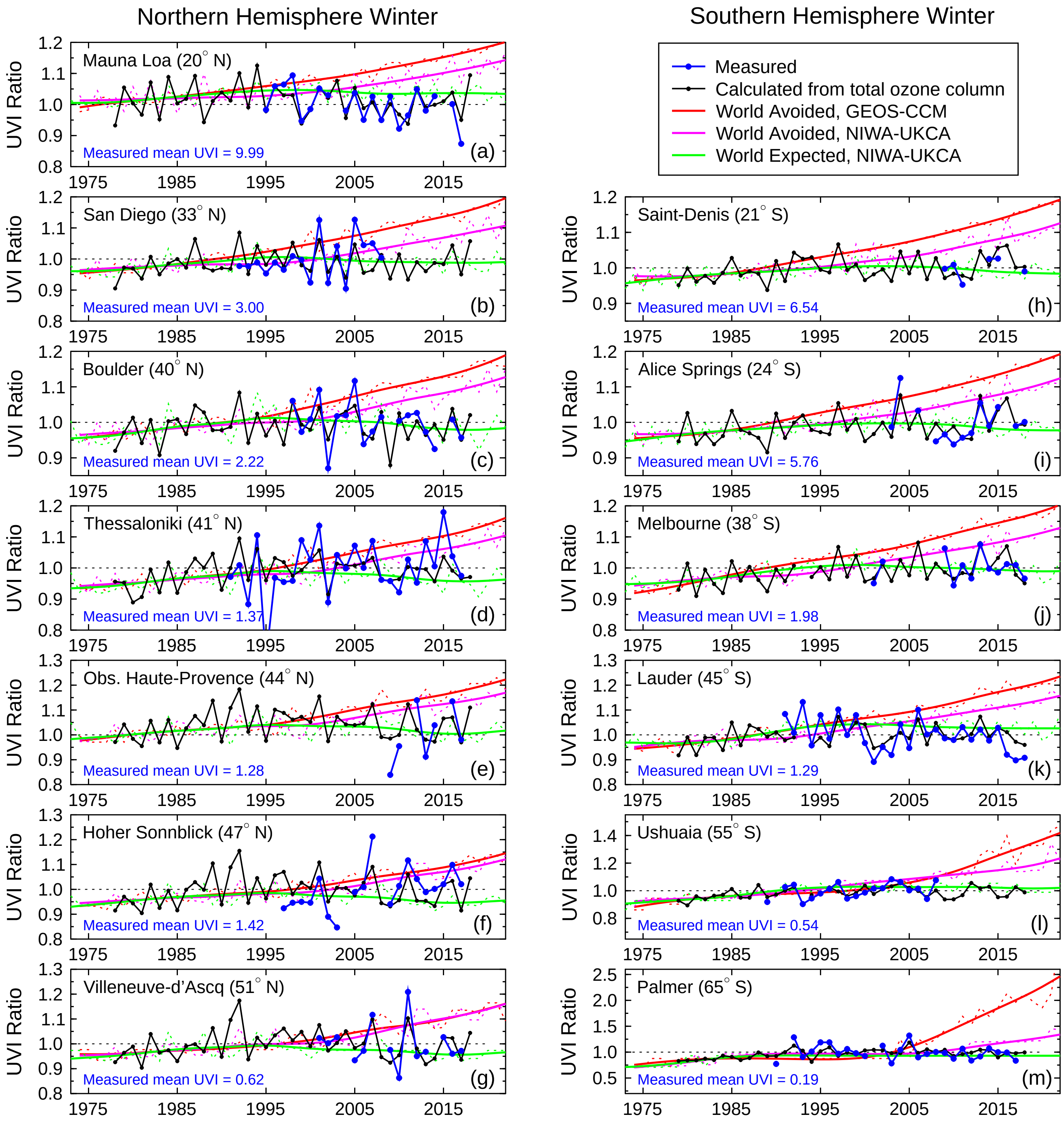
Northern Hemisphere Winter
Southern Hemisphere Winter
Measured
Calculated from total ozone column
World Avoided, GEOS-CCM
World Avoided, NIWA-UKCA
World Expected, NIWA-UKCA
UVI Ratio
Mauna Loa (20° N)
Measured mean UVI = 9.99
(a)
San Diego (33° N)
Measured mean UVI = 3.00
(b)
Boulder (40° N)
Measured mean UVI = 2.22
(c)
Thessaloniki (41° N)
Measured mean UVI = 1.37
(d)
Obs. Haute-Provence (44° N)
Measured mean UVI = 1.28
(e)
Hoher Sonnblick (47° N)
Measured mean UVI = 1.42
(f)
Villeneuve-d'Ascq (51° N)
Measured mean UVI = 0.62
(g)
Saint-Denis (21° S)
Measured mean UVI = 6.54
(h)
Alice Springs (24° S)
Measured mean UVI = 5.76
(i)
Melbourne (38° S)
Measured mean UVI = 1.98
(j)
Lauder (45° S)
Measured mean UVI = 1.29
(k)
Ushuaia (55° S)
Measured mean UVI = 0.54
(l)
Palmer (65° S)
Measured mean UVI = 0.19
(m)
1975
1985
1995
2005
2015

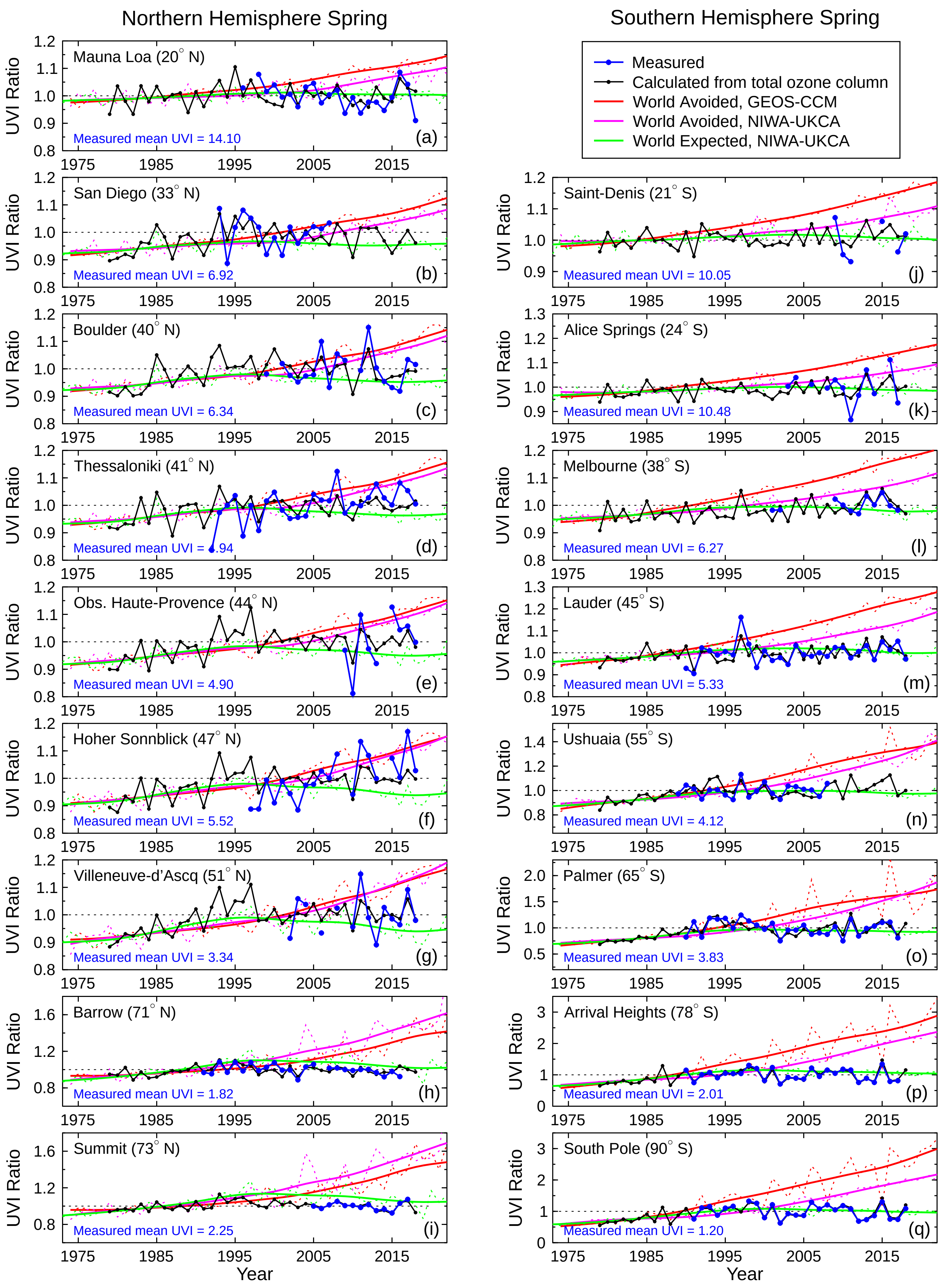

Northern Hemisphere Spring
Southern Hemisphere Spring
Measured
Calculated from total ozone column
World Avoided, GEOS-CCM
World Avoided, NIWA-UKCA
World Expected, NIWA-UKCA
Mauna Loa (20° N)
Measured mean UVI = 14.10
(a)
San Diego (33° N)
Measured mean UVI = 6.92
(b)
Boulder (40° N)
Measured mean UVI = 6.34
(c)
Thessaloniki (41° N)
Measured mean UVI = 4.94
(d)
Obs. Haute-Provence (44° N)
Measured mean UVI = 4.90
(e)
Hoher Sonnblick (47° N)
Measured mean UVI = 5.52
(f)
Villeneuve-d'Ascq (51° N)
Measured mean UVI = 3.34
(g)
Barrow (71° N)
Measured mean UVI = 1.82
(h)
Summit (73° N)
Measured mean UVI = 2.25
(i)
Saint-Denis (21° S)
Measured mean UVI = 10.05
(j)
Alice Springs (24° S)
Measured mean UVI = 10.48
(k)
Melbourne (38° S)
Measured mean UVI = 6.27
(l)
Lauder (45° S)
Measured mean UVI = 5.33
(m)
Ushuaia (55° S)
Measured mean UVI = 4.12
(n)
Palmer (65° S)
Measured mean UVI = 3.83
(o)
Arrival Heights (78° S)
Measured mean UVI = 2.01
(p)
South Pole (90° S)
Measured mean UVI = 1.20
(q)
UVI Ratio
Year
1975
1985
1995
2005
2015

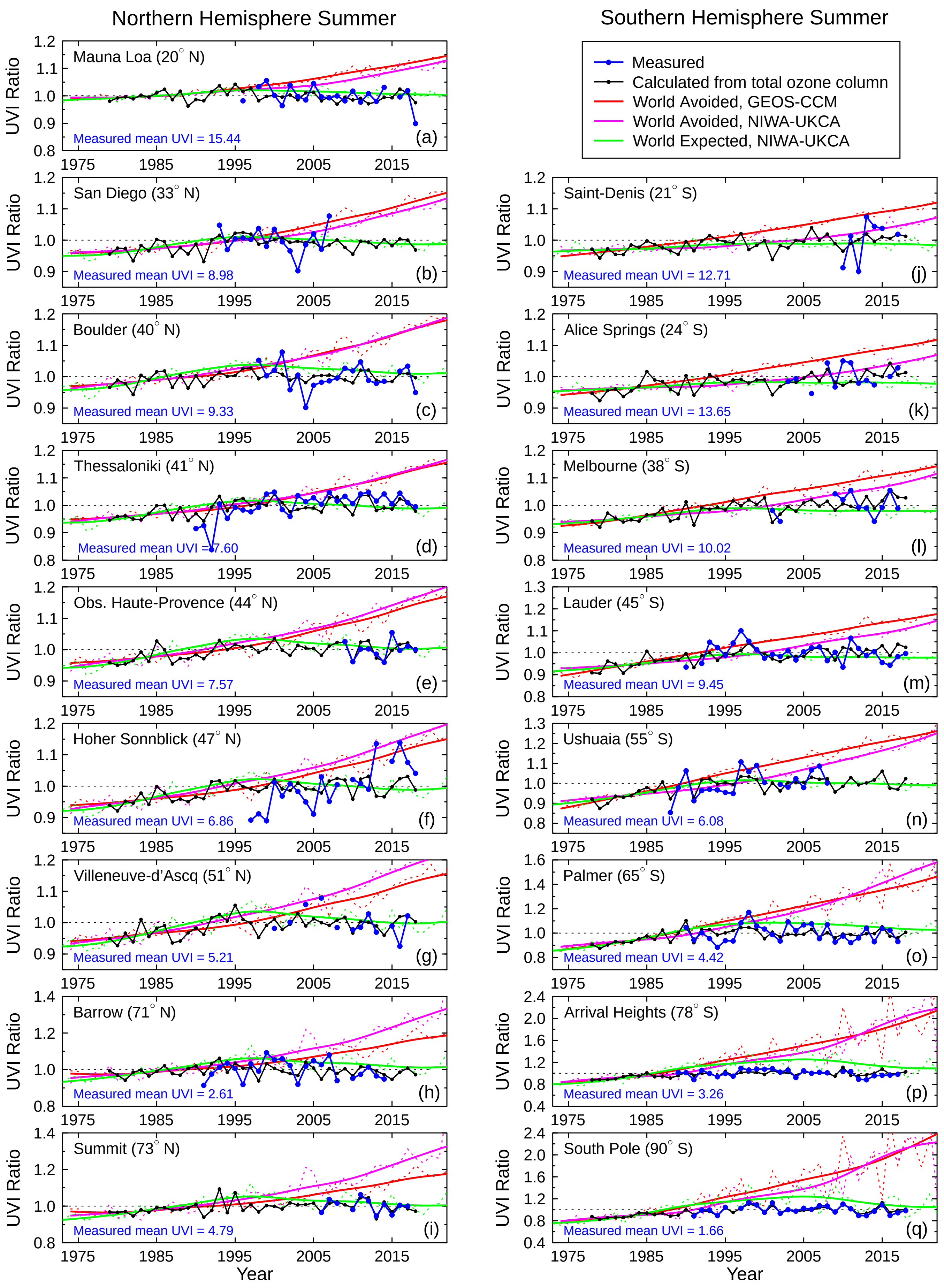

Northern Hemisphere Summer
Southern Hemisphere Summer
Measured
Calculated from total ozone column
World Avoided, GEOS-CCM
World Avoided, NIWA-UKCA
World Expected, NIWA-UKCA
Mauna Loa (20° N)
Measured mean UVI = 15.44
(a)
San Diego (33° N)
Measured mean UVI = 8.98
(b)
Boulder (40° N)
Measured mean UVI = 9.33
(c)
Thessaloniki (41° N)
Measured mean UVI = 7.60
(d)
Obs. Haute-Provence (44° N)
Measured mean UVI = 7.57
(e)
Hoher Sonnblick (47° N)
Measured mean UVI = 6.86
(f)
Villeneuve-d'Ascq (51° N)
Measured mean UVI = 5.21
(g)
Barrow (71° N)
Measured mean UVI = 2.61
(h)
Summit (73° N)
Measured mean UVI = 4.79
(i)
Saint-Denis (21° S)
Measured mean UVI = 12.71
(j)
Alice Springs (24° S)
Measured mean UVI = 13.65
(k)
Melbourne (38° S)
Measured mean UVI = 10.02
(l)
Lauder (45° S)
Measured mean UVI = 9.45
(m)
Ushuaia (55° S)
Measured mean UVI = 6.08
(n)
Palmer (65° S)
Measured mean UVI = 4.42
(o)
Arrival Heights (78° S)
Measured mean UVI = 3.26
(p)
South Pole (90° S)
Measured mean UVI = 1.66
(q)
UVI Ratio
Year
1975
1985
1995
2005
2015

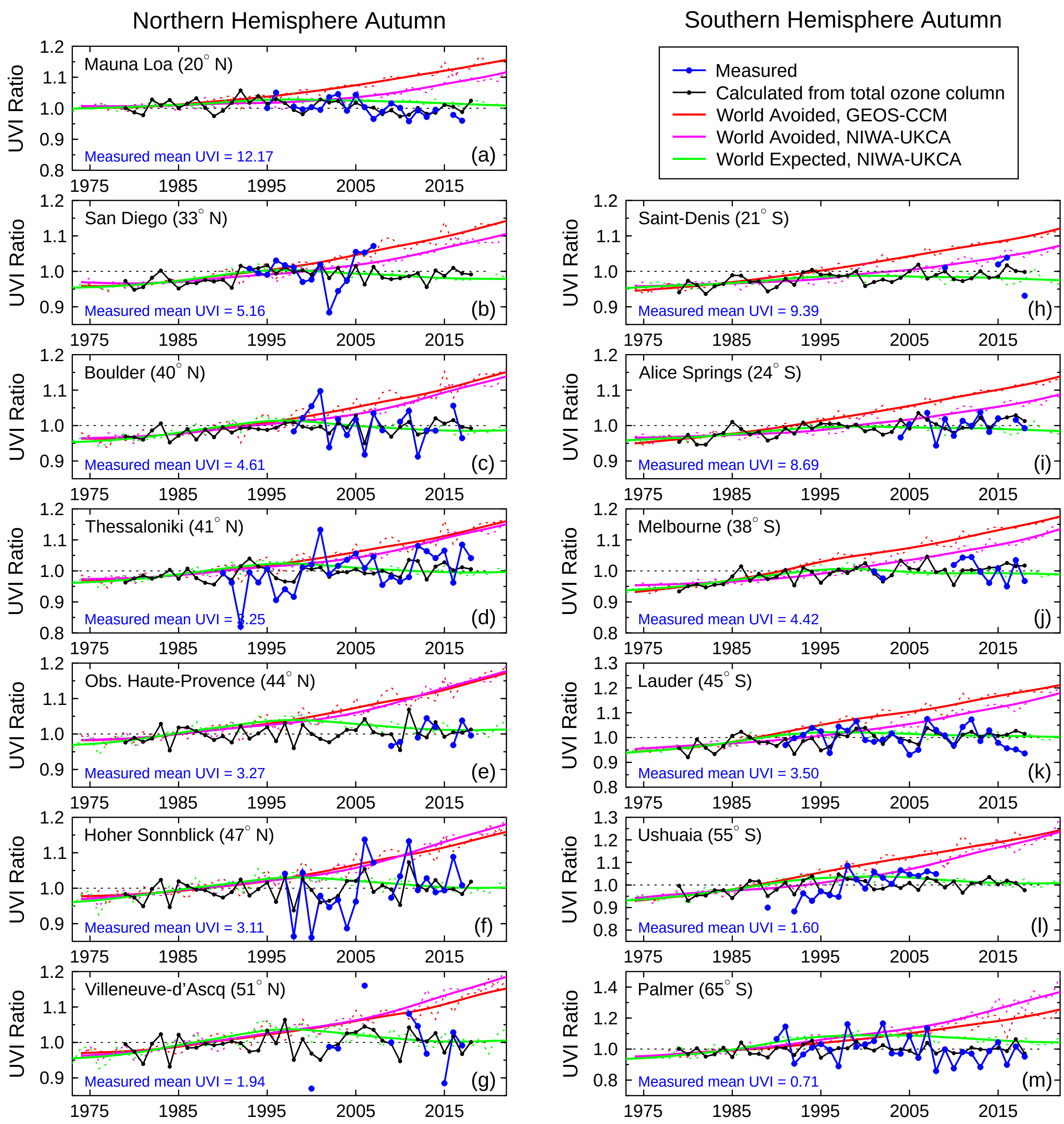
Northern Hemisphere Autumn
Southern Hemisphere Autumn
Measured
Calculated from total ozone column
World Avoided, GEOS-CCM
World Avoided, NIWA-UKCA
World Expected, NIWA-UKCA
UVI Ratio
Mauna Loa (20° N)
Measured mean UVI = 12.17
(a)
San Diego (33° N)
Measured mean UVI = 5.16
(b)
Boulder (40° N)
Measured mean UVI = 4.61
(c)
Thessaloniki (41° N)
(d)
Obs. Haute-Provence (44° N)
Measured mean UVI = 3.27
(e)
Hoher Sonnblick (47° N)
Measured mean UVI = 3.11
(f)
Villeneuve-d'Ascq (51° N)
Measured mean UVI = 1.94
(g)
Saint-Denis (21° S)
Measured mean UVI = 9.39
(h)
Alice Springs (24° S)
Measured mean UVI = 8.69
(i)
Melbourne (38° S)
Measured mean UVI = 4.42
(j)
Lauder (45° S)
Measured mean UVI = 3.50
(k)
Ushuaia (55° S)
Measured mean UVI = 1.60
(l)
Palmer (65° S)
Measured mean UVI = 0.71
(m)
1975
1985
1995
2005
2015
